# Supplementary figures and images for: Ionic-liquid-facilitated transdermal absorption of lidocaine hydrochloride
Source: PLoS One. 2026 Apr 3;21(4):e0344789. doi: 10.1371/journal.pone.0344789 (PMC13048389; doi:10.1371/journal.pone.0344789)

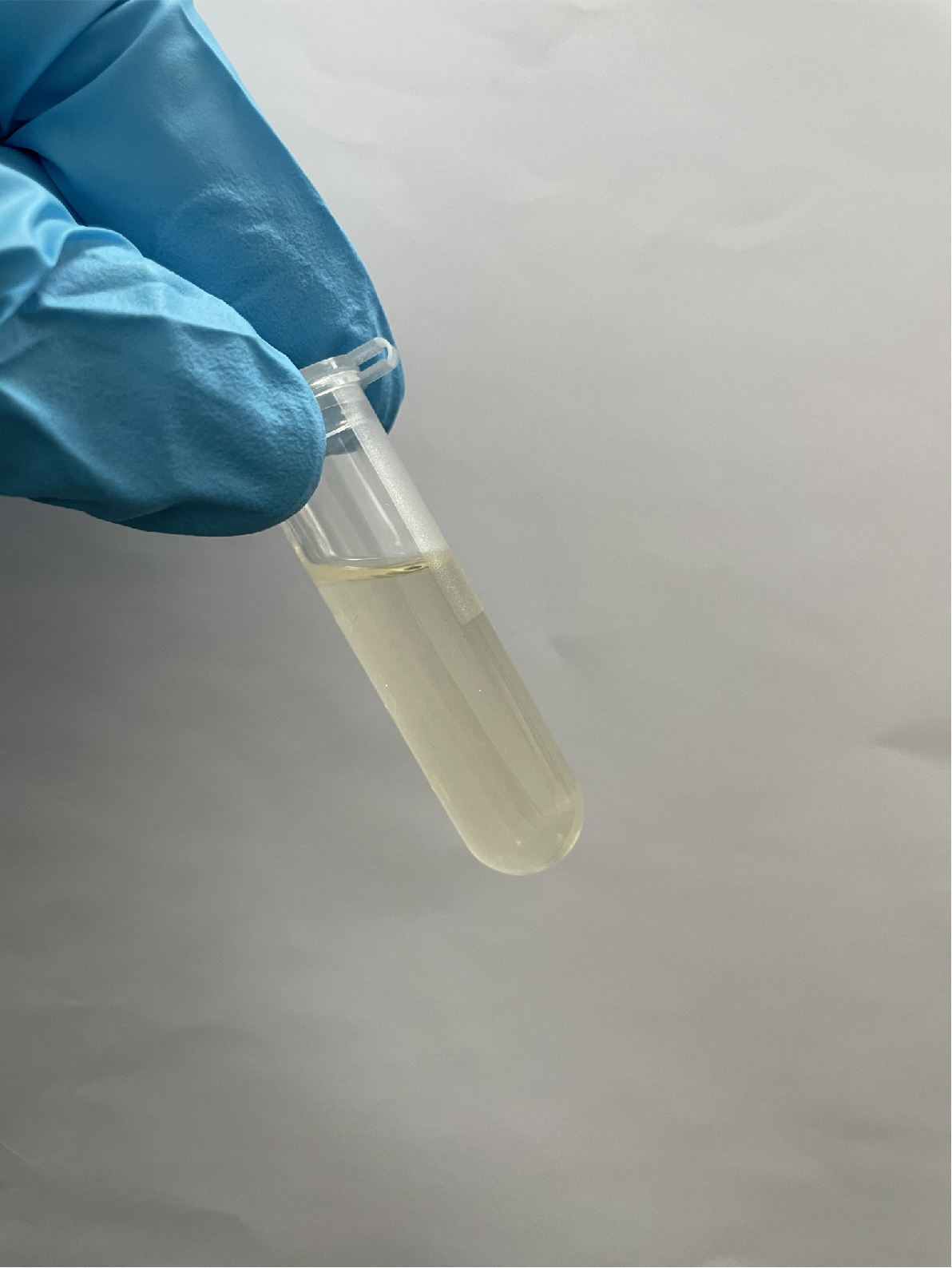

Supplement: S1 Fig — The IL presents as a clear and slightly yellow solution. (TIF) [file pone.0344789.s001.tif]

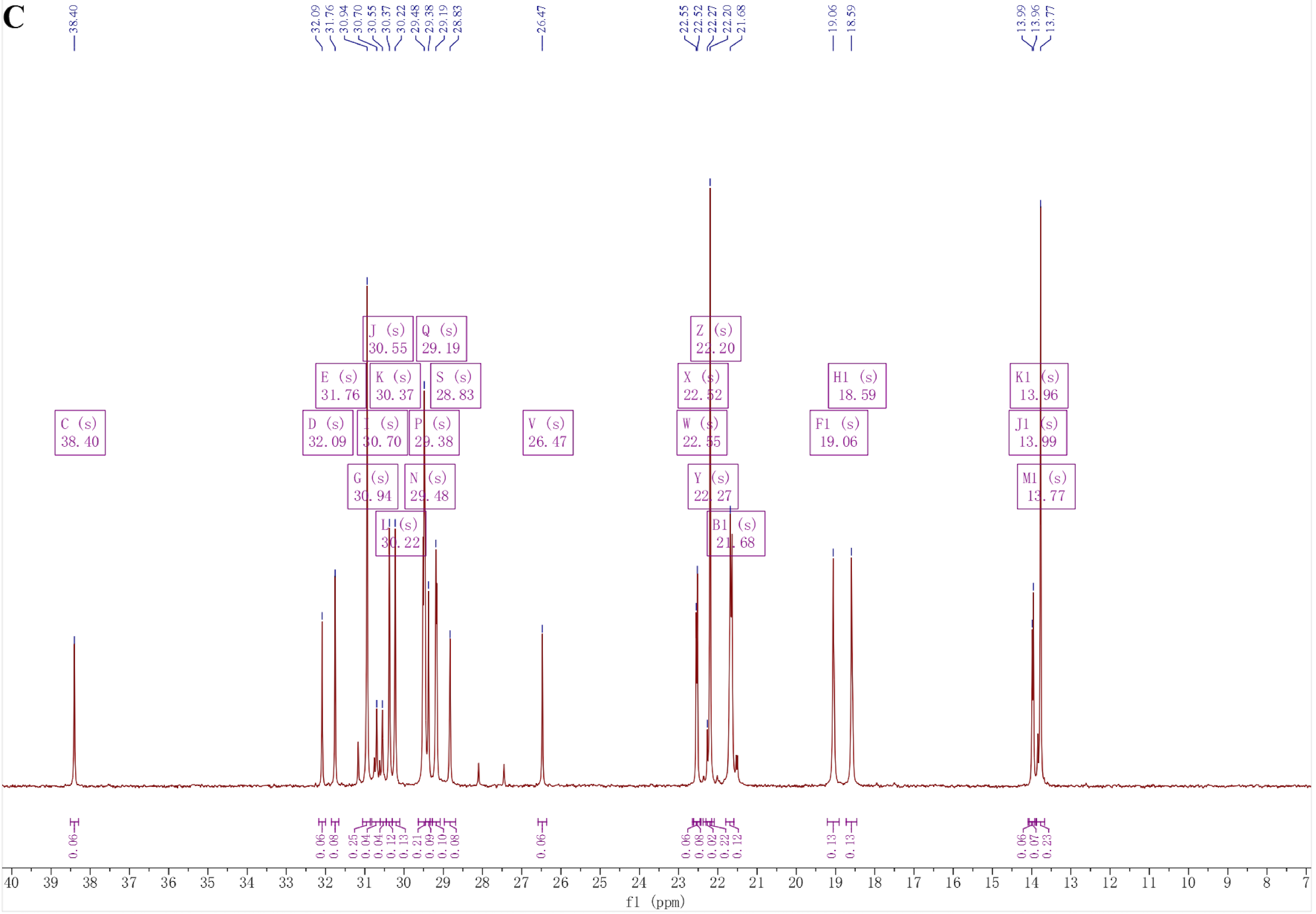

Supplement: S2 Fig — The characteristic resonance peaks observed in the spectrum are consistent with those reported in previous studies, which confirms the successful synthesis and structural integrity of the IL. (TIF) [file pone.0344789.s002.tif]
