# Supplementary material for: Ionic-liquid-facilitated transdermal absorption of lidocaine hydrochloride
Source: PLoS One. 2026 Apr 3;21(4):e0344789. doi: 10.1371/journal.pone.0344789 (PMC13048389; doi:10.1371/journal.pone.0344789)
Supplement: Attachment — Submitted filename: PONE-D-25-63253_with comments_01282026.pdf [file pone.0344789.s006.pdf]

PLOS One

Ionic-liquid-facilitated transdermal absorption of lidocaine hydrochloride

--Manuscript Draft--

**Manuscript Number:**	PONE-D-25-63253R1
**Article Type:**	Research Article
**Full Title:**	Ionic-liquid-facilitated transdermal absorption of lidocaine hydrochloride
**Short Title:**	Ionic Liquid Lidocaine Delivery
**Corresponding Author:**	Yuqi Wen, M.D.<br>Central South University First Hospital: Xiangya Hospital Central South University<br>Changsha, CHINA
**Keywords:**	lidocaine hydrochloride, ionic liquid, transdermal absorption,analgesic delivery
**Abstract:**	The increasing worldwide need for analgesics, along with patients' aversion to injectable anesthetics and limited compliance, highlights the necessity for noninvasive local anesthesia. However, the skin barrier poses a significant obstacle to the efficient transdermal administration of anesthetics. In this study, we synthesized a new ionic liquid (IL), tetradecylphosphonium hexanoate, by mixing sodium hexanoate and CYPHOS 101 at equimolar ratios. We systematically evaluated the capacity of this IL to improve the transdermal transfer of lidocaine hydrochloride through in vitro skin permeation tests and cytotoxicity investigations in HaCaT cells. The IL significantly enhanced the percutaneous absorption of lidocaine in a concentration-dependent manner. At 80 mM, the permeation efficiency increased by approximately 1.5 times compared with the control group, resulting in a significantly reduced onset time for topical anesthesia. While IL showed minimal cytotoxicity at low concentrations, higher concentrations were linked to increased cytotoxicity. ILs can effectively enhance the permeation of transdermal lidocaine. The ease of synthesizing ILs, their adjustable properties, and good compatibility make them promising for creating advanced topical anesthetics and broadening transdermal drug delivery applications.
**Order of Authors:**	Xiaoting Ma
	Yangzhe Zhou
	Ke Jiang
	Yuqi Wen, M.D.
**Response to Reviewers:**	Response to Reviewers<br><br>Manuscript ID: PONE-D-25-63253<br>Title: Ionic-liquid-facilitated transdermal absorption of lidocaine hydrochloride<br><br>Dear Academic Editor and Reviewers,<br><br>Thank you for the opportunity to revise our manuscript and for the constructive comments provided by the reviewers. We have carefully addressed all points raised and have made substantial revisions to improve the manuscript. Our point-by-point responses are detailed below. All changes in the manuscript have been highlighted using the "Track Changes" function.<br><br>Responses to Reviewer #1:<br><br>We sincerely thank Reviewer #1 for the thorough review and valuable suggestions.<br><br>1. Comment: The introduction contains a conclusion statement. "Skin permeation tests conducted in vitro confirmed…" should not appear in the introduction.<br>Response: We agree and have revised the final paragraph of the Introduction section. The conclusive statements have been removed and replaced with a clear statement of the study's aims and hypothesis.<br><br>2. Comment: Please consider adding a picture of the Franz diffusion cell setup.

Powered by Editorial Manager® and ProduXion Manager® from Aries Systems Corporation

Response: A schematic diagram of the Franz diffusion cell setup is already provided as Fig. 1 in the original submission. We have now extended the title of this chart.

3. Comment: The sex of SD rats should be specified to exclude potential hormonal influences.
Response: We have specified that male SD rats were used in the revised Section 2.3 (Animal preparation).

4. Comment: Please clarify why skin samples were stored at −20 °C and used within two weeks.
Response: An explanation has been added to Section 2.3: skin samples were stored at −20 °C to preserve the integrity of the skin barrier and were used within two weeks to ensure experimental consistency in the in vitro permeation studies.

5. Comment: Does this study include in vivo results?
Response: This study is limited to in vitro evaluations. We have acknowledged this as a key limitation in the new Section 4. Discussion (Limitations and future perspectives).

6. Comment: Please provide ED50/LD50 data from the toxicity test.
Response: The cytotoxicity assay was conducted on HaCaT cells to assess in vitro biocompatibility and was not designed to determine in vivo lethal doses (LD50). We have clarified this point in the Section 2.7 (Cytotoxicity test) and in the Discussion.

7. Comment: It is better to separate the Results and Discussion sections.
Response: As suggested, we have restructured the manuscript by separating the combined “Results and discussion” section into two distinct chapters: “3. Results” and “4. Discussion.”

Responses to Reviewer #2:

We sincerely thank Reviewer #2 (Dr. Shashank Bhangde) for their thorough review, positive feedback, and constructive suggestions, which have significantly strengthened our manuscript.

1. Comment: Consider adding statistical analysis to the data where possible.
Response: We have performed comprehensive statistical analysis as suggested. A new Section 2.8 (Statistical analysis) has been added, describing the use of one-way ANOVA followed by Tukey’s post hoc test. The error bars in Fig. 3 and Fig. 4 now represent the standard deviation (SD), and statistically significant differences are indicated with asterisks (**$p < 0.01$, ***$p < 0.001$). The results sections (3.3 and 3.4) now include explicit statements of statistical significance.

2. Comment: The specific term “analgesic drug delivery” was missing.
Response: We thank the reviewer for highlighting this important omission. To accurately frame the therapeutic focus of our work and enhance the manuscript’s discoverability, we have added “analgesic drug delivery” as a keyword in the revised manuscript.

3. Comment: All citations appear at the end of the statement.
Response: We have reviewed and revised the manuscript to ensure all citations are placed at the end of their respective statements for consistency, including in the Introduction where general statements about lidocaine and ionic liquids were lacking support.

4. Comment: In current clinical setting, anesthesia is giving SC. You have only shown 1.5x increase in comparison to lidocaine given transdermally (which is not clinical standard). Rephrase this line.
Response: We thank the reviewer for this crucial clarification. We have rephrased the relevant line in the Conclusions section。

5. Comment: General suggestion to add future research directions.
Response: We appreciate this suggestion. As guided by the Editor, we have expanded the Conclusions to include a specific sentence on future work: “Future

	studies should focus on the formulation development of a practical transdermal delivery system and the preclinical evaluation of lidocaine bioavailability in vivo."<br><br>6. Comment: "I have a little flow issue. The flow should be: synthesis – detection – cytotoxicity – skin permeation."<br>Response: We agree with the reviewer's suggestion for a more logical narrative flow. In the revised manuscript, we have reorganized the "3. Results" section accordingly:<br>3.1 Characterization of the IL<br>3.2 Detection of lidocaine hydrochloride<br>3.3 HaCaT cell viability evaluation of the IL<br>3.4 In vitro skin permeation assays<br>We believe this new order—presenting the material, then its safety profile, followed by its functional efficacy—provides a clearer and more compelling argument. All in-text citations to figures and results have been updated to reflect this new structure.We have also reordered the corresponding sections in '2. Materials and methods' to maintain consistency with this improved narrative flow.<br><br>7. Comment:The issue of inconsistent units (ml, mg, μg)<br>Response: We thank the reviewer for identifying the unit errors. All instances of incorrect or ambiguous units have been identified and standardized throughout the manuscript to ensure accuracy and consistency.<br><br>Additional Revisions in Response to Journal Requirements:<br><br>We have also addressed all points raised by the Academic Editor to ensure full compliance with PLOS ONE policies.<br><br>1. Style Requirements: The manuscript has been adjusted as per the requirements and the structure has been ensured to comply with the guidelines.<br>2. ORCID iD: The corresponding author's ORCID iD is registered and validated in the submission system.<br>3. Animal Ethics Details: As requested, Section 2.3 has been expanded to include specific details on (1) the method of euthanasia (anesthetic overdose), (2) the method of anesthesia (intraperitoneal injection of pentobarbital sodium, 50 mg/kg), and (3) efforts to minimize suffering (all procedures performed under deep anesthesia).<br>4. NMR Data: Details regarding the NMR instrument (Bruker 400 MHz), magnetic field strength have been added to Section 2.2. The NMR spectrum is provided as Fig. S2 in the Supporting Information<br>5. & 6. & 7. Funding Information: As instructed, all funding-related text has been removed from the manuscript's Acknowledgements section. The funding statement provided in the submission system remains accurate. The role of the funders is stated here, as requested for the cover letter: "The funders were involved in providing financial support for the experiment and in the decision to publish."<br>8. & 9. Supporting Information: Captions for the Supporting Information files (Fig. S1 and Fig. S2) have been included at the end of the main manuscript file. The file types have been designated correctly in the submission system.<br><br>We believe the revised manuscript has been significantly improved and now fully meets the publication standards of PLOS ONE. We are grateful for the insightful comments and hope you find our revisions satisfactory.<br><br>Sincerely,<br><br>Yangzhe Zhou, Yuqi Wen<br>On behalf of all co-authors
**Additional Information:**	
**Question**	**Response**
**Financial Disclosure**<br><br>Enter a financial disclosure statement that describes the sources of funding for the	Yes

work included in this submission. Review the submission guidelines for detailed requirements. View published research articles from *PLOS ONE* for specific examples.<br><br>This statement is required for submission and **will appear in the published article** if the submission is accepted. Please make sure it is accurate.<br><br>**Funded studies**<br>Enter a statement with the following details:<br>• Initials of the authors who received each award<br>• Grant numbers awarded to each author<br>• The full name of each funder<br>• URL of each funder website<br>• Did the sponsors or funders play any role in the study design, data collection and analysis, decision to publish, or preparation of the manuscript?<br><br>Did you receive funding for this work?	
Please add funding details.<br>as follow-up to "**Financial Disclosure**<br><br>Enter a financial disclosure statement that describes the sources of funding for the work included in this submission. Review the submission guidelines for detailed requirements. View published research articles from *PLOS ONE* for specific examples.<br><br>This statement is required for submission and **will appear in the published article** if the submission is accepted. Please make sure it is accurate.	This work was supported by the National Science Foundation of China (8207104259), the Hunan Provincial Science and Technology Plan Project, China (No. 2019TP1001), and the Innovation Driven Project of Central South University (2020CX002).

**Funded studies**<br>Enter a statement with the following details:<br>• Initials of the authors who received each award<br>• Grant numbers awarded to each author<br>• The full name of each funder<br>• URL of each funder website<br>• Did the sponsors or funders play any role in the study design, data collection and analysis, decision to publish, or preparation of the manuscript?<br><br>Did you receive funding for this work?"	
Please select the country of your main research funder (please select carefully as in some cases this is used in fee calculation).<br>as follow-up to "**Financial Disclosure**<br><br>Enter a financial disclosure statement that describes the sources of funding for the work included in this submission. Review the submission guidelines for detailed requirements. View published research articles from *PLOS ONE* for specific examples.<br><br>This statement is required for submission and **will appear in the published article** if the submission is accepted. Please make sure it is accurate.<br><br>**Funded studies**<br>Enter a statement with the following details:<br>• Initials of the authors who received each award<br>• Grant numbers awarded to each author<br>• The full name of each funder<br>• URL of each funder website<br>• Did the sponsors or funders play any role in the study design, data collection and analysis, decision to publish, or preparation of the manuscript?<br><br>Did you receive funding for this work?"	CHINA - CN
**Competing Interests**	The authors have declared that no competing interests exist.

Use the instructions below to enter a competing interest statement for this submission. On behalf of all authors, disclose any competing interests that could be perceived to bias this work—acknowledging all financial support and any other relevant financial or non-financial competing interests.<br><br>This statement is required for submission and **will appear in the published article** if the submission is accepted. Please make sure it is accurate and that any funding sources listed in your Funding Information later in the submission form are also declared in your Financial Disclosure statement.<br><br>View published research articles from *PLOS ONE* for specific examples.<br><br>**NO authors have competing interests**<br><br>Enter: *The authors have declared that no competing interests exist.*<br><br>**Authors with competing interests**<br><br>Enter competing interest details beginning with this statement:<br><br>*I have read the journal's policy and the authors of this manuscript have the following competing interests: [insert competing interests here]*<br><br>* typeset	
**Ethics Statement**<br><br>Enter an ethics statement for this submission. This statement is required if the study involved:<br><br>• Human participants<br>• Human specimens or tissue<br>• Vertebrate animals or cephalopods<br>• Vertebrate embryos or tissues	Male SD rats (weight: 180–220 g, age: 7–8 weeks) were provided by the Xiangya School of Medicine, Central South University(ethics audit number: CSU-2022-0170). All animals were housed under standard conditions (room temperature, 12-h light/dark cycle) with free access to a standard pellet diet and water. After a one-week acclimatization period, the rats were deeply anesthetized by an intraperitoneal injection of pentobarbital sodium (50 mg/kg).Euthanasia was subsequently performed via an overdose of the same anesthetic, and death was confirmed by the absence of breathing and cardiac arrest.The abdominal hair was then shaved, and the full-thickness abdominal skin was surgically excised. All subcutaneous fat and connective tissue were carefully removed. The excised skin samples were stored at −20 °C in a

• Field research<br><br>Write "N/A" if the submission does not require an ethics statement.<br><br>General guidance is provided below. Consult the submission guidelines for detailed instructions. **Make sure that all information entered here is included in the Methods section of the manuscript.**	freezer and used within two weeks to preserve the integrity of the skin barrier for consistent in vitro permeation studies. The experiment was conducted in compliance with the Code of Ethics of the World Medical Association and approved by the Experimental Animal Welfare and Ethics Committee of Xiangya School of Medicine, Central South University.

Powered by Editorial Manager® and ProduXion Manager® from Aries Systems Corporation

**Format for specific study types**<br><br>**Human Subject Research (involving human participants and/or tissue)**<br>• Give the name of the institutional review board or ethics committee that approved the study<br>• Include the approval number and/or a statement indicating approval of this research<br>• Indicate the form of consent obtained (written/oral) or the reason that consent was not obtained (e.g. the data were analyzed anonymously)<br><br>**Animal Research (involving vertebrate animals, embryos or tissues)**<br>• Provide the name of the Institutional Animal Care and Use Committee (IACUC) or other relevant ethics board that reviewed the study protocol, and indicate whether they approved this research or granted a formal waiver of ethical approval<br>• Include an approval number if one was obtained<br>• If the study involved *non-human primates*, add *additional details* about animal welfare and steps taken to ameliorate suffering<br>• If anesthesia, euthanasia, or any kind of animal sacrifice is part of the study, include briefly which substances and/or methods were applied<br><br>**Field Research**<br><br>Include the following details if this study involves the collection of plant, animal, or other materials from a natural setting:<br>• Field permit number<br>• Name of the institution or relevant body that granted permission	
**Data Availability**<br><br>Authors are required to make all data underlying the findings described fully available, without restriction, and from the time of publication. PLOS allows rare exceptions to address legal and ethical concerns. See the PLOS Data Policy and FAQ for detailed information.	Yes - all data are fully available without restriction

A Data Availability Statement describing where the data can be found is required at submission. Your answers to this question constitute the Data Availability Statement and **will be published in the article**, if accepted.<br><br>**Important:** Stating 'data available on request from the author' is not sufficient. If your data are only available upon request, select 'No' for the first question and explain your exceptional situation in the text box.<br><br>Do the authors confirm that all data underlying the findings described in their manuscript are fully available without restriction?	
**Describe where the data may be found in full sentences. If you are copying our sample text, replace any instances of XXX with the appropriate details.**<br><br>• If the data are **held or will be held in a public repository**, include URLs, accession numbers or DOIs. If this information will only be available after acceptance, indicate this by ticking the box below. For example: *All XXX files are available from the XXX database (accession number(s) XXX, XXX.*).<br>• If the data are all contained **within the manuscript and/or Supporting Information files**, enter the following: *All relevant data are within the manuscript and its Supporting Information files.*<br>• If neither of these applies but you are able to provide **details of access elsewhere**, with or without limitations, please do so. For example:<br><br>*Data cannot be shared publicly because of [XXX]. Data are available from the XXX Institutional Data Access / Ethics Committee (contact via XXX) for researchers who meet the criteria for access to confidential data.*<br><br>*The data underlying the results presented in the study are available from (include the name of the third party*	All relevant data are within the manuscript and its Supporting Information files.

and contact information or URL).<br>• This text is appropriate if the data are owned by a third party and authors do not have permission to share the data.<br><br>* typeset	
Additional data availability information:	

Powered by Editorial Manager® and ProduXion Manager® from Aries Systems Corporation

Manuscript Click here to access/download;Manuscript;Manuscript.docx

Ionic-liquid-facilitated transdermal absorption of lidocaine hydrochloride

Xiaoting Ma[a], Yangzhe Zhou[b,*], Ke Jiang[a], Yuqi Wen[a,*]

[a] Department of Traditional Chinese Medicine, Xiangya Hospital, Central South University, Changsha 410000, China
[b] Department of Plastic surgery, Xiangya Hospital, Central South University, Changsha 410000, China

* Corresponding authors Email: zo7858667@gmail.com (Y. Zhou)
* Corresponding authors Email: wenyuqi1221@163.com (Y. Wen)

Abstract

The increasing worldwide need for analgesics, along with patients' aversion to injectable anesthetics and limited compliance, highlights the necessity for noninvasive local anesthesia. However, the skin barrier poses a significant obstacle to the efficient transdermal administration of anesthetics. In this study, we synthesized a new ionic liquid (IL), tetradecylphosphonium hexanoate, by mixing sodium hexanoate and CYPHOS 101 at equimolar ratios. We systematically evaluated the capacity of this IL to improve the transdermal transfer of lidocaine hydrochloride through in vitro skin permeation tests and cytotoxicity investigations in HaCaT cells. The IL significantly enhanced the percutaneous absorption of lidocaine in a concentration-dependent manner. At 80 mM, the permeation efficiency increased by approximately 1.5 times compared with the control group, resulting in a significantly reduced onset time for topical anesthesia. While IL showed minimal cytotoxicity at low concentrations, higher concentrations were linked to increased cytotoxicity. ILs can effectively enhance the permeation of transdermal lidocaine. The ease of synthesizing ILs, their adjustable properties, and good compatibility make them promising for creating advanced topical anesthetics and broadening transdermal drug delivery applications.

Keywords: lidocaine hydrochloride, ionic liquid, transdermal absorption, analgesic delivery

1. Introduction

Pain can result from various diseases and injuries, significantly reducing quality

of life and imposing substantial economic and social burdens (1-6). For instance, the global prevalence of low back pain was estimated at 619 million in 2020, projected to reach 843 million by 2050, consistently ranking it as the primary cause of disability burden (7). Lidocaine hydrochloride is a commonly used amide-type local anesthetic in clinical practice and serves as a Class Ib antiarrhythmic agent for treating ventricular arrhythmias. Its primary mechanism involves blocking voltage-dependent sodium channels, thereby inhibiting the generation and conduction of action potentials in nerves or cardiac muscles (8). In anesthesiology, lidocaine provides a rapid onset, good penetration, and effective surface anesthesia for various clinical scenarios, including infiltration, nerve block, and surface/mucosal anesthesia. Its potency and duration fall within a moderate range among commonly used local anesthetics, with favorable tissue diffusion owing to its physicochemical properties. Upon absorption into the bloodstream or intravenous administration, it exerts distinct dual excitatory and inhibitory effects on the central nervous system (8-13).

The most common clinical administration route for lidocaine hydrochloride is subcutaneous injection. However, this method is often accompanied by significant stinging and local discomfort, thereby reducing patient compliance (14-17). To overcome this limitation, researchers have developed various alternative delivery strategies, including ultrasound-mediated transdermal delivery, electroporation, gel formulations, microneedles, and transdermal delivery (18-31). Compared with other routes, transdermal delivery is considered advantageous owing to its noninvasiveness and avoidance of the first-pass effect, significantly improving drug bioavailability and

patient experience (12, 26, 32). However, the skin stratum corneum—the body's primary natural barrier—remains the main obstacle limiting effective transdermal drug absorption (33).

In recent years, ionic liquids (ILs) have attracted significant attention due to their distinct physicochemical properties and remarkable capacity to enhance skin permeability(34-39). Comprising specific ratios of organic cations to organic/inorganic anions, IL typically possess melting points below 100 °C and remain liquid at room temperature, displaying outstanding solubility, thermal stability, and adjustability (34-39). These attributes hold great promise for drug delivery applications. Mandal et al. significantly enhanced the transdermal delivery and therapeutic effectiveness of NFKBIZ siRNA by combining it with IL for psoriasis treatment (40). Banerjee et al. utilized IL to enhance the oral absorption efficiency of insulin, presenting new opportunities for diabetes management (41). Therefore, ILs are not only innovative transdermal permeation enhancers but also poised to serve as a vital platform for delivering complex drugs and biologics in the future.

In this study, we aimed to utilize a novel IL, tetradecylphosphonium hexanoate, synthesized from CYPHOS 101 and sodium hexanoate, to enhance the transdermal absorption of lidocaine hydrochloride. We hypothesized that this IL would disrupt the stratum corneum barrier and increase drug permeation while maintaining acceptable biocompatibility. To test this hypothesis, we systematically evaluated the permeation-enhancing efficacy through in vitro skin permeation assays and assessed preliminary safety via cytotoxicity studies in HaCaT keratinocytes.

2. Materials and methods

2.1 Materials

Sodium hexanoate (99% purity) was acquired from Aladdin. Trihexyltetradecylphosphonium (tetraalkylphosphonium) chloride (CYPHOS 101, purity ≥ 97%), lidocaine hydrochloride (purity: 99%), and silver nitrate solution were procured from Macklin. Phosphate buffer for cell culture was purchased from Procell. Dimethyl sulfoxide (DMSO) and MTT for cell proliferation assays were sourced from SolarBio. HaCaT cells were obtained from Xiangya Hospital of Central South University. All reagents were of analytical grade and used without additional purification.

2.2 Instruments

Transdermal testing utilized a Franz diffusion cell (TK-12D, Shanghai Kaikai). Characterization was performed using nuclear magnetic resonance (NMR) on a Bruker 400 MHz (Switzerland). Ultraviolet–visible (UV–vis) absorption spectra were obtained with a UV-2450 spectrophotometer (SHIMADZU, Japan). The Elx800 microplate reader (BioTek, U.S) was utilized.

2.3 Animal preparation

Male SD rats (weight: 180–220 g, age: 7–8 weeks) were provided by the Xiangya School of Medicine, Central South University(ethics audit number: CSU-2022-0170). All animals were housed under standard conditions (room temperature,

12-h light/dark cycle) with free access to a standard pellet diet and water. After a one-week acclimatization period, the rats were deeply anesthetized by an intraperitoneal injection of pentobarbital sodium (50 mg/kg). Euthanasia was subsequently performed via an overdose of the same anesthetic, and death was confirmed by the absence of breathing and cardiac arrest. The abdominal hair was then shaved, and the full-thickness abdominal skin was surgically excised. All subcutaneous fat and connective tissue were carefully removed. The excised skin samples were stored at −20 °C in a freezer and used within two weeks to preserve the integrity of the skin barrier for consistent in vitro permeation studies. The experiment was conducted in compliance with the Code of Ethics of the World Medical Association and approved by the Experimental Animal Welfare and Ethics Committee of Xiangya School of Medicine, Central South University.

2.4 Synthesis of the IL

First, sodium hexanoate (1.46 g, 0.011 mol) powder was dissolved in 15 mL of methanol. CYPHOS 101 (5.48 g, 0.011 mol) was then added to the solution and stirred for 15–20 min until fully mixed. Methanol was removed by rotary evaporation, and the IL was washed with deionized water until the washed water was chloride-free. IL was subsequently dried in a vacuum oven at 80 °C for 48 h. For the preparation of the lidocaine hydrochloride-IL, lidocaine hydrochloride powder was added to the IL to achieve a concentration of 1 mg/mL and thoroughly mixed.

2.5 Detection of lidocaine hydrochloride

Lidocaine hydrochloride was prepared at concentrations of 2, 3, 4, 6, 8, and 10

μg/mL. Subsequently, 100 μL of each concentration was introduced into the cuvette for analysis with a UV spectrometer.

2.6 Cytotoxicity test

To evaluate the cytotoxicity of IL, HaCaT cells were selected as the model due to their suitability in mimicking the human epidermal state (42). First, 1×10^4 cells in 100 μL of culture medium were seeded into each well of a 96-well plate and incubated overnight. Upon confirming cell adhesion, the culture medium was aspirated and replaced with varying concentrations of IL (30, 50, and 80 mM) for 5 h. Subsequently, the medium was exchanged with 100 μL of Dulbecco's modified Eagle's medium (DMEM) and incubated for an additional 19 h (24 h in total). The control group was treated solely with DMEM throughout the experiment. Following this, 20 μL of MTT solution (0.5 mg/mL) was added to each well and incubated for 4 h. After discarding the MTT solution, 150 μL of DMSO was introduced, and the plate was agitated for 20 min on a balanced shaker. The absorbance was measured at 490 nm using a microplate reader.This assay evaluated in vitro cellular biocompatibility and was not designed to determine in vivo lethal dose (LD50) parameters.

2.7 In vitro skin permeation assays

The IL at concentrations of 30, 50, and 80 mM was combined with lidocaine hydrochloride (at a fixed concentration of 1 mg/mL) and assessed for transdermal absorption efficiency. The transdermal testing apparatus, as illustrated in Fig. 1, consisted of a donor chamber positioned at the top, SD mouse skin treated in the middle (effective diffusion area: 3.14 cm^2), and an acceptor chamber at the bottom

with a capacity of 8 mL.

First, a small magnet was positioned in the acceptor chamber, with skin placed between the acceptor and donor chambers. The stratum corneum (SC) of the skin was elevated, and rat skin was slightly stretched to minimize wrinkles. Subsequently, 8 mL of phosphate-buffered saline (PBS) was added to the acceptor chamber. Particular attention was paid to the discharge of subcutaneous air bubbles; otherwise, the experimental results would have been affected. Then, 3 mL of IL/lidocaine hydrochloride solution with different IL concentrations was added to each donor chamber. The donor chamber was sealed with a film containing small holes to reduce evaporation. At regular intervals, 200 μL of the sample was removed from the sampling port and supplemented with 200 μL of PBS to keep the volume of the acceptor chamber constant. The chamber is placed in a magnetic box at a constant temperature of 37 °C for experiments.

Fig 1.Schematic of the Franz diffusion cell setup for in vitro skin permeation studies. Schematic illustration of the Franz diffusion cell used for transdermal permeation experiments, showing donor, receptor, and skin membrane compartments.

2.8 Sstatistical analysis

Data are presented as mean ± standard deviation (SD) of at least three independent experiments. For skin permeation and cell viability studies, statistical significance was determined using one-way analysis of variance (ANOVA) followed by Tukey's post hoc test for multiple comparisons. A p-value of less than 0.05 was considered statistically significant. All analyses were performed using GraphPad

Prism software.

3. Results

3.1 Characterization of the IL

The synthesized IL appeared as a clear, slightly yellow solution (Supporting Information, Fig. S1). The NMR spectrum of the IL was consistent with previous reports (Supporting Information, Fig. S2) (43): 1H NMR (400 MHz, Chloroform-d) δ 2.53 (s, 1H), 2.43 (ddt, J = 13.0, 9.6, 5.4 Hz, 2H), 1.63 (dd, J = 17.3, 5.4 Hz, 0H), 1.47 (t, J = 4.9 Hz, 0H), 1.45–1.25 (m, 7H), 0.87 (d, J = 3.3 Hz, 0H); and 13C NMR (101 MHz, Chloroform-d) δ 179.22, 38.40, 32.09, 31.76, 30.94, 30.70, 30.55, 30.37, 30.22, 29.48, 29.38, 29.19, 28.83, 26.47, 22.55, 22.52, 22.27, 22.20, 21.68, 19.06, 18.59, 13.99, 13.96, and 13.77.

3.2 Detection of lidocaine hydrochloride

As shown in Fig. 2, the absorbance intensity of the lidocaine hydrochloride solution at approximately 196 nm increased with increasing lidocaine hydrochloride concentrations within the 2–10 μg/mL range, exhibiting a correlation coefficient of 0.999. These absorbance readings were subsequently employed in a subsequent experiment to evaluate the transdermal administration of lidocaine hydrochloride.

Fig 2.UV–Vis calibration of lidocaine hydrochloride. UV absorption spectra of lidocaine hydrochloride at different concentrations (a–f: 2, 3, 4, 6, 8, and 10 μg mL^{-1}) and calibration curve showing the linear relationship between absorbance at 196 nm and lidocaine concentration

3.3 HaCaT cell viability evaluation of the IL

The cytotoxicity of the IL towards HaCaT keratinocytes was evaluated, and the results are presented in Fig. 3. The cell viability relative to the untreated control was 89.3 ± 1.9% for the 30 mM IL group and 88.9 ± 3.1% for the 50 mM IL group. There was no statistically significant difference between these two groups ($p > 0.05$, Tukey's test). In contrast, exposure to 80 mM IL significantly reduced cell viability to 41.2 ± 0.8% of the control level ($p < 0.001$ versus the control, 30 mM, and 50 mM groups; one-way ANOVA with Tukey's post hoc test).

Fig 3.Cytotoxicity of ionic liquids in HaCaT cells. Viability of HaCaT cells treated with different concentrations of ionic liquids (ILs). After 5 h of IL exposure, the medium was replaced with fresh DMEM and cells were incubated for an additional 19 h (24 h total). Cell viability was assessed using the MTT assay. Data are presented as mean ± SD ($n = 5$). ***$p < 0.001$ versus the control group (one-way ANOVA with Tukey's post hoc test).

3.4 In vitro skin permeation assays

As shown in Fig. 4, the cumulative permeation of lidocaine hydrochloride increased in a time and IL concentration-dependent manner. The permeation approached a plateau after approximately 4 hours. At the 5-hour time point, the cumulative amount permeated in the presence of 80 mM IL was approximately 1.5-fold higher than that in the control group (without IL), and this difference was statistically significant ($p < 0.05$). Both 50 mM and 80 mM IL groups showed significant enhancement compared to the control ($p < 0.05$) from the 3-hour time

point onward, with no significant difference between these two IL groups observed within the first 3 hours.

Fig 4.Effect of ionic liquid concentration on lidocaine permeation. Enhanced transdermal absorption efficiency of lidocaine hydrochloride in the presence of different concentrations of ionic liquids (ILs). Data points represent the mean ± SD (n = 3). Asterisks indicate statistically significant differences compared to the control group at the corresponding time point (**p < 0.01, ***p < 0.001, one-way ANOVA with Tukey's post hoc test).

4. Discussion

This study demonstrates that the synthesized IL significantly enhanced the transdermal permeation of lidocaine hydrochloride in a concentration-dependent manner. The 1.5-fold increase in cumulative permeation achieved with 80 mM IL suggests a strong potential to shorten the onset time of topical anesthesia, addressing a key clinical need for rapid-acting, non-invasive analgesics. Critically, we identified 50 mM as an optimal concentration for further development. At this level, the IL provided substantial permeation enhancement while maintaining high viability in HaCaT cells, indicating a favorable balance between efficacy and biocompatibility. This concentration is particularly relevant given that the acceptable waiting time for topical anesthesia in clinical practice is typically under one hour, a duration within which the 50 mM formulation demonstrates effective delivery. However, these promising in vitro findings are subject to limitations. Future work must include in vivo models to validate the anesthetic efficacy and systemic safety, employ more

complex skin models (e.g., reconstructed human epidermis) for comprehensive safety assessment, and investigate the formulation's long-term stability. Despite these necessary next steps, the current results establish a solid foundation for the development of advanced IL-facilitated transdermal drug delivery systems.

5. Conclusions

In this study, a novel tetraalkylphosphonium hexanoate IL was successfully synthesized. In vitro evaluations demonstrated that this IL significantly enhanced the transdermal permeation of lidocaine hydrochloride in a concentration-dependent manner, with an approximately 1.5-fold increase observed at 80 mM compared to the control. Importantly, at the effective concentration of 50 mM, the IL exhibited minimal cytotoxicity against HaCaT keratinocytes. These findings collectively support the potential of this IL as an effective and biocompatible permeation enhancer for topical lidocaine delivery.Future studies should focus on the formulation development of a practical transdermal delivery system and the preclinical evaluation of lidocaine bioavailability in vivo.

Acknowledgements

The authors thank colleagues for their technical assistance and valuable discussions.

Author contributions:

Yuqi Wen, Xiaoting Ma, Yangzhe Zhou: Conception and design of the study; Acquisition of data; Analysis and interpretation of data; Provision of reagents, materials, analysis tools, or data; Drafting the manuscript. Ke Jiang: Conception and design of the study; Analysis and interpretation of data.

Competing interests

The authors declare no competing interests.

Data availability

All data generated or analysed during this study are included in this published article (and its Supplementary Information files).

References

1. Cagle JG, Carr DC, Hong S, Zimmerman S. Financial burden among US households affected by cancer at the end of life. Psychooncology. 2016;25(8):919-26.
2. Fitzcharles MA, Cohen SP, Clauw DJ, Littlejohn G, Usui C, Häuser W. Nociplastic pain: towards an understanding of prevalent pain conditions. Lancet. 2021;397(10289):2098-110.
3. Jiang C, Wang H, Wang Q, Luo Y, Sidlow R, Han X. Prevalence of Chronic Pain and High-Impact Chronic Pain in Cancer Survivors in the United States. JAMA Oncol. 2019;5(8):1224-6.
4. Paice JA. Cancer pain management and the opioid crisis in America: How to preserve hard-earned gains in improving the quality of cancer pain management. Cancer. 2018;124(12):2491-7.
5. Shkodra M, Brunelli C, Zecca E, Formaglio F, Bracchi P, Lo Dico S, et al. Neuropathic pain: clinical classification and assessment in patients with pain due to cancer. Pain. 2021;162(3):866-74.
6. van den Beuken-van Everdingen MH, Hochstenbach LM, Joosten EA, Tjan-Heijnen VC, Janssen DJ. Update on Prevalence of Pain in Patients With Cancer: Systematic Review and Meta-Analysis. J Pain Symptom Manage. 2016;51(6):1070-90.e9.
7. Global, regional, and national burden of low back pain, 1990-2020, its attributable risk factors, and projections to 2050: a systematic analysis of the Global Burden of Disease Study 2021. Lancet Rheumatol. 2023;5(6):e316-e29.

8. Taylor A, McLeod G. Basic pharmacology of local anaesthetics. BJA Educ. 2020;20(2):34-41.
9. Bahar E, Yoon H. Lidocaine: A Local Anesthetic, Its Adverse Effects and Management. Medicina (Kaunas). 2021;57(8).
10. Beaussier M, Delbos A, Maurice-Szamburski A, Ecoffey C, Mercadal L. Perioperative Use of Intravenous Lidocaine. Drugs. 2018;78(12):1229-46.
11. de Carvalho JF, Skare TL. Lidocaine in fibromyalgia: A systematic review. World J Psychiatry. 2022;12(4):615-22.
12. Wang Y, Su W, Li Q, Li C, Wang H, Li Y, et al. Preparation and evaluation of lidocaine hydrochloride-loaded TAT-conjugated polymeric liposomes for transdermal delivery. Int J Pharm. 2013;441(1-2):748-56.
13. Zhang D, Ye D, Jing P, Tan X, Qiu L, Li T, et al. Design, optimization and evaluation of co-surfactant free microemulsion-based hydrogel with low surfactant for enhanced transdermal delivery of lidocaine. Int J Pharm. 2020;586:119415.
14. Chu CC, Wu SZ, Su WL, Shieh JP, Kao CH, Ho ST, et al. Subcutaneous injection of inhaled anesthetics produces cutaneous analgesia. Can J Anaesth. 2008;55(5):290-4.
15. Ellis BD, Kosmorsky GS. Referred ocular pain relieved by suboccipital injection. Headache. 1995;35(2):101-3.
16. Klein JA, Jeske DR. Estimated Maximal Safe Dosages of Tumescent Lidocaine. Anesth Analg. 2016;122(5):1350-9.
17. Levin M. Nerve blocks in the treatment of headache. Neurotherapeutics. 2010;7(2):197-203.
18. Batheja P, Thakur R, Michniak B. Transdermal iontophoresis. Expert Opin Drug Deliv. 2006;3(1):127-38.
19. Ito Y, Ohta J, Imada K, Akamatsu S, Tsuchida N, Inoue G, et al. Dissolving microneedles to obtain rapid local anesthetic effect of lidocaine at skin tissue. J Drug Target. 2013;21(8):770-5.
20. Kathuria H, Li H, Pan J, Lim SH, Kochhar JS, Wu C, et al. Large Size Microneedle Patch to Deliver Lidocaine through Skin. Pharm Res. 2016;33(11):2653-67.
21. Kochhar JS, Lim WX, Zou S, Foo WY, Pan J, Kang L. Microneedle integrated transdermal patch for fast onset and sustained delivery of lidocaine. Mol Pharm. 2013;10(11):4272-80.
22. Li Y, Zhao E, Li L, Bai L, Zhang W. Facile design of lidocaine-loaded polymeric hydrogel to persuade effects of local anesthesia drug delivery system: complete in vitro and in vivo toxicity analyses. Drug Deliv. 2021;28(1):1080-92.
23. Liu DZ, Sheu MT, Chen CH, Yang YR, Ho HO. Release characteristics of lidocaine from local implant of polyanionic and polycationic hydrogels. J Control Release. 2007;118(3):333-9.
24. Long J, Etxeberria AE, Nand AV, Bunt CR, Ray S, Seyfoddin A. A 3D printed chitosan-pectin hydrogel wound dressing for lidocaine hydrochloride delivery. Mater Sci Eng C Mater Biol Appl. 2019;104:109873.
25. Nayak A, Babla H, Han T, Das DB. Lidocaine carboxymethylcellulose with gelatine co-polymer hydrogel delivery by combined microneedle and ultrasound. Drug Deliv. 2016;23(2):658-69.
26. Prausnitz MR, Langer R. Transdermal drug delivery. Nat Biotechnol. 2008;26(11):1261-8.
27. Rasool A, Ata S, Islam A, Rizwan M, Azeem MK, Mehmood A, et al. Kinetics and controlled release of lidocaine from novel carrageenan and alginate-based blend hydrogels. Int J Biol Macromol. 2020;147:67-78.

28. Sammeta SM, Vaka SR, Murthy SN. Transdermal drug delivery enhanced by low voltage electropulsation (LVE). Pharm Dev Technol. 2009;14(2):159-64.
29. Soto F, Jeerapan I, Silva-López C, Lopez-Ramirez MA, Chai I, Xiaolong L, et al. Noninvasive Transdermal Delivery System of Lidocaine Using an Acoustic Droplet-Vaporization Based Wearable Patch. Small. 2018;14(49):e1803266.
30. Tachibana K, Tachibana S. Use of ultrasound to enhance the local anesthetic effect of topically applied aqueous lidocaine. Anesthesiology. 1993;78(6):1091-6.
31. Wallace MS, Ridgeway B, Jun E, Schulteis G, Rabussay D, Zhang L. Topical delivery of lidocaine in healthy volunteers by electroporation, electroincorporation, or iontophoresis: an evaluation of skin anesthesia. Reg Anesth Pain Med. 2001;26(3):229-38.
32. Yuan JS, Ansari M, Samaan M, Acosta EJ. Linker-based lecithin microemulsions for transdermal delivery of lidocaine. Int J Pharm. 2008;349(1-2):130-43.
33. Ahad A, Aqil M, Kohli K, Chaudhary H, Sultana Y, Mujeeb M, et al. Chemical penetration enhancers: a patent review. Expert Opin Ther Pat. 2009;19(7):969-88.
34. Curreri AM, Mitragotri S, Tanner EEL. Recent Advances in Ionic Liquids in Biomedicine. Adv Sci (Weinh). 2021;8(17):e2004819.
35. Egorova KS, Gordeev EG, Ananikov VP. Biological Activity of Ionic Liquids and Their Application in Pharmaceutics and Medicine. Chem Rev. 2017;117(10):7132-89.
36. Lei Z, Chen B, Koo YM, MacFarlane DR. Introduction: Ionic Liquids. Chem Rev. 2017;117(10):6633-5.
37. Qi QM, Mitragotri S. Mechanistic study of transdermal delivery of macromolecules assisted by ionic liquids. J Control Release. 2019;311-312:162-9.
38. Tanner EEL, Curreri AM, Balkaran JPR, Selig-Wober NC, Yang AB, Kendig C, et al. Design Principles of Ionic Liquids for Transdermal Drug Delivery. Adv Mater. 2019;31(27):e1901103.
39. Zakrewsky M, Lovejoy KS, Kern TL, Miller TE, Le V, Nagy A, et al. Ionic liquids as a class of materials for transdermal delivery and pathogen neutralization. Proc Natl Acad Sci U S A. 2014;111(37):13313-8.
40. Mandal A, Kumbhojkar N, Reilly C, Dharamdasani V, Ukidve A, Ingber DE, et al. Treatment of psoriasis with NFKBIZ siRNA using topical ionic liquid formulations. Sci Adv. 2020;6(30):eabb6049.
41. Banerjee A, Ibsen K, Brown T, Chen R, Agatemor C, Mitragotri S. Ionic liquids for oral insulin delivery. Proc Natl Acad Sci U S A. 2018;115(28):7296-301.
42. Cui D, Bi J, Zhang ZN, Li MY, Qin YS, Xiang P, et al. Organophosphorus flame retardant TDCPP-induced cytotoxicity and associated mechanisms in normal human skin keratinocytes. Sci Total Environ. 2020;726:138526.
43. Oster K, Goodrich P, Jacquemin J, Hardacre C, Ribeiro APC, Elsinawi A. A new insight into pure and water-saturated quaternary phosphonium-based carboxylate ionic liquids: Density, heat capacity, ionic conductivity, thermogravimetric analysis, thermal conductivity and viscosity. The Journal of Chemical Thermodynamics. 2018;121:97-111.

Supporting information

S1 Fig Appearance of the synthesized ionic liquid (IL) Photograph showing the

physical state of the synthesized ionic liquid. The IL presents as a clear and slightly yellow solution.

S2 Fig Nuclear Magnetic Resonance (NMR) spectrum of the synthesized ionic liquid (IL) NMR spectrum of the target synthesized ionic liquid. The characteristic resonance peaks observed in the spectrum are consistent with those reported in previous studies, which confirms the successful synthesis and structural integrity of the IL.

Click here to access/download;Figure;Fig 1.tif

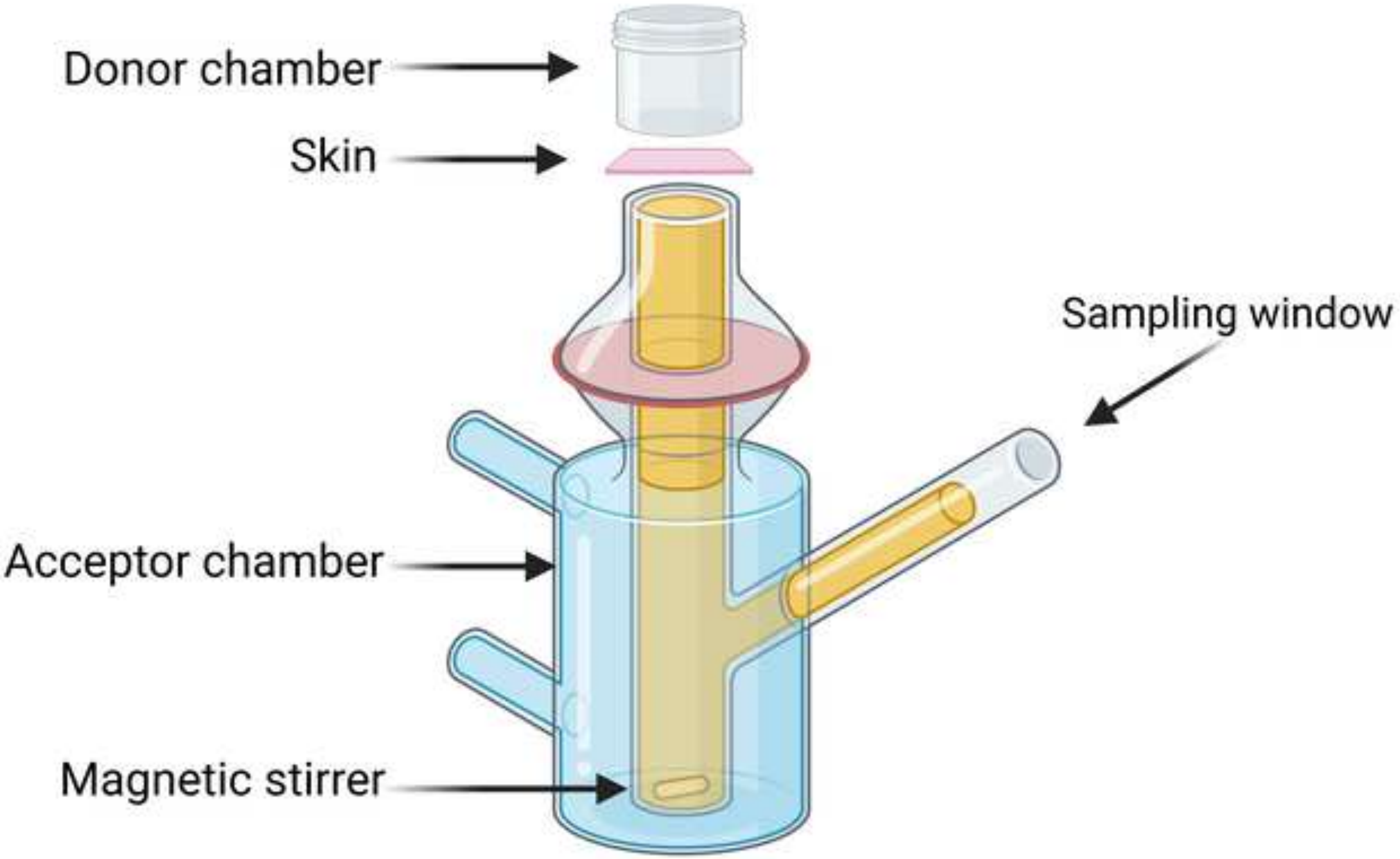

Click here to access/download;Figure;Fig 2.tif

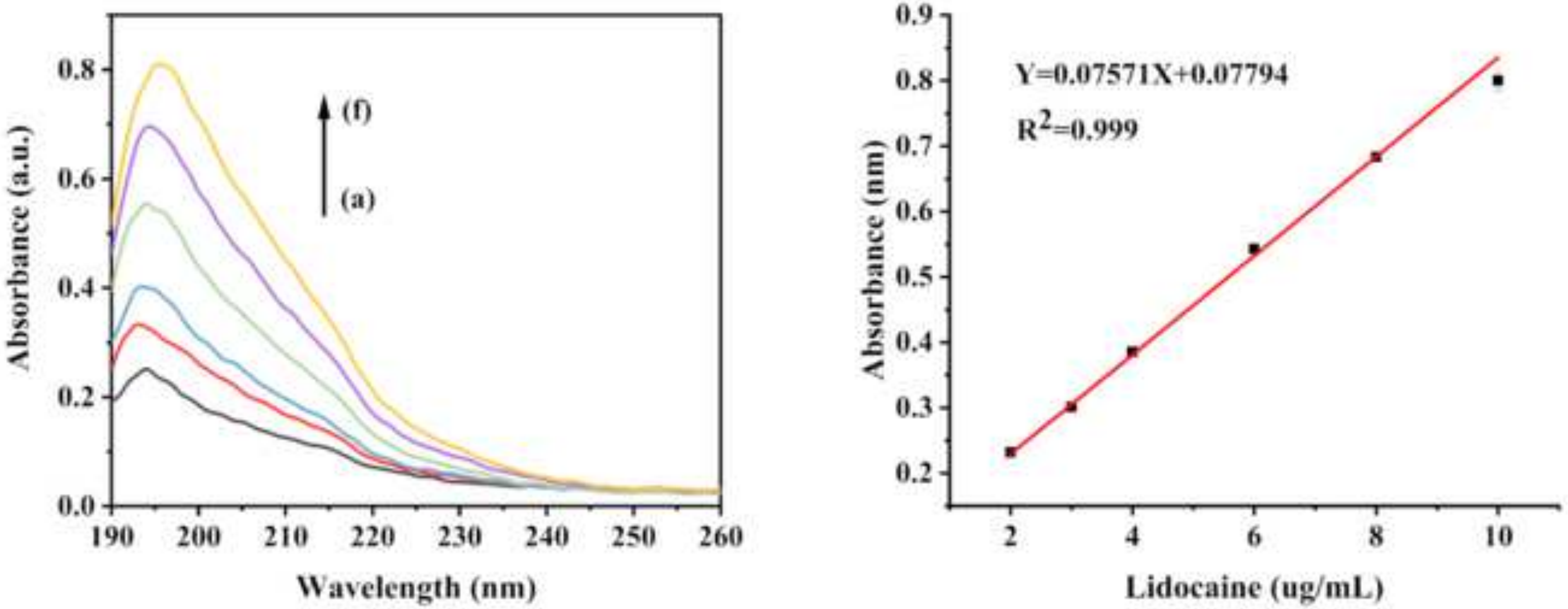

Click here to access/download;Figure;Fig 3.tiff

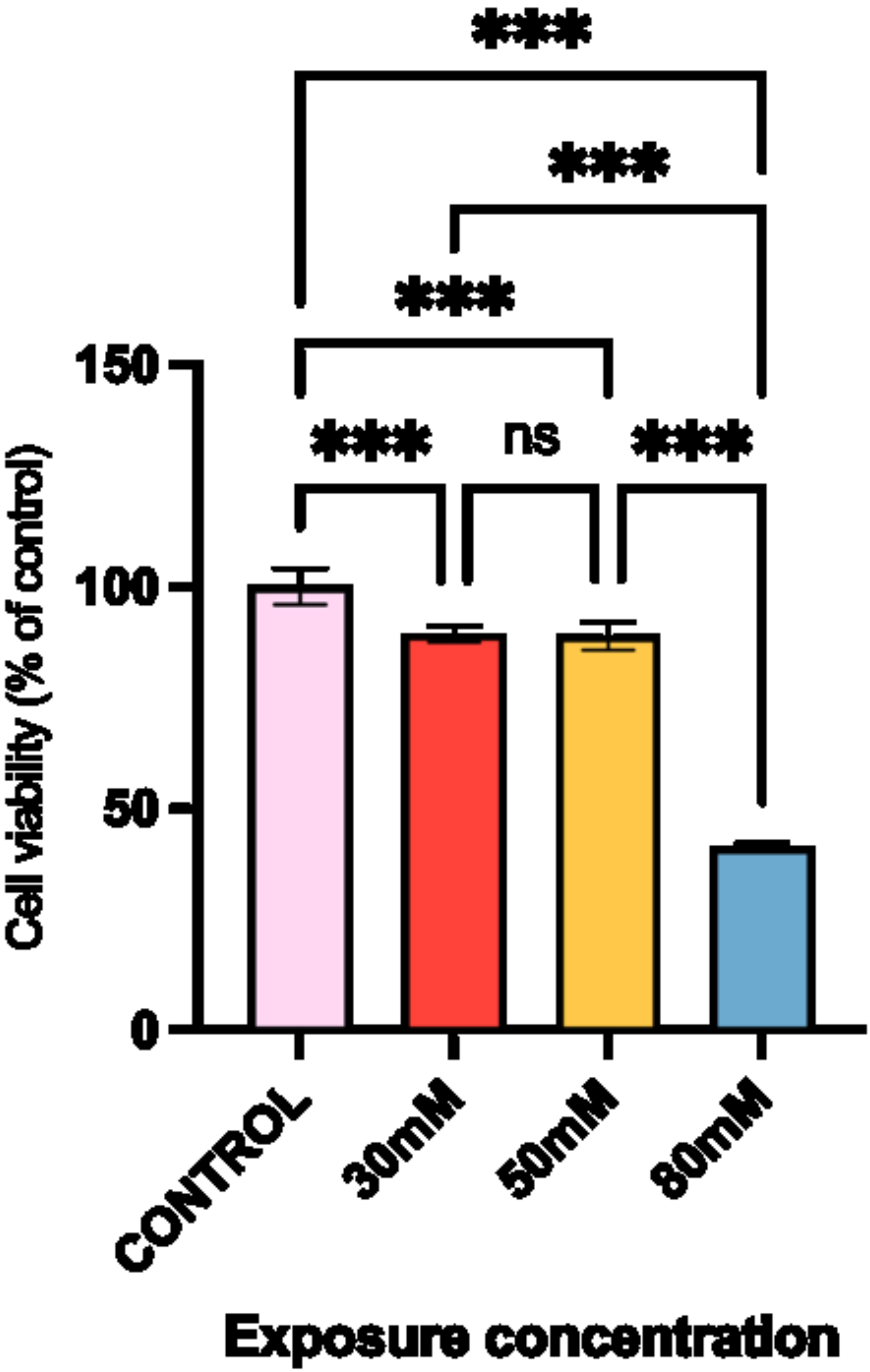

Click here to access/download;Figure;Fig 4.tif

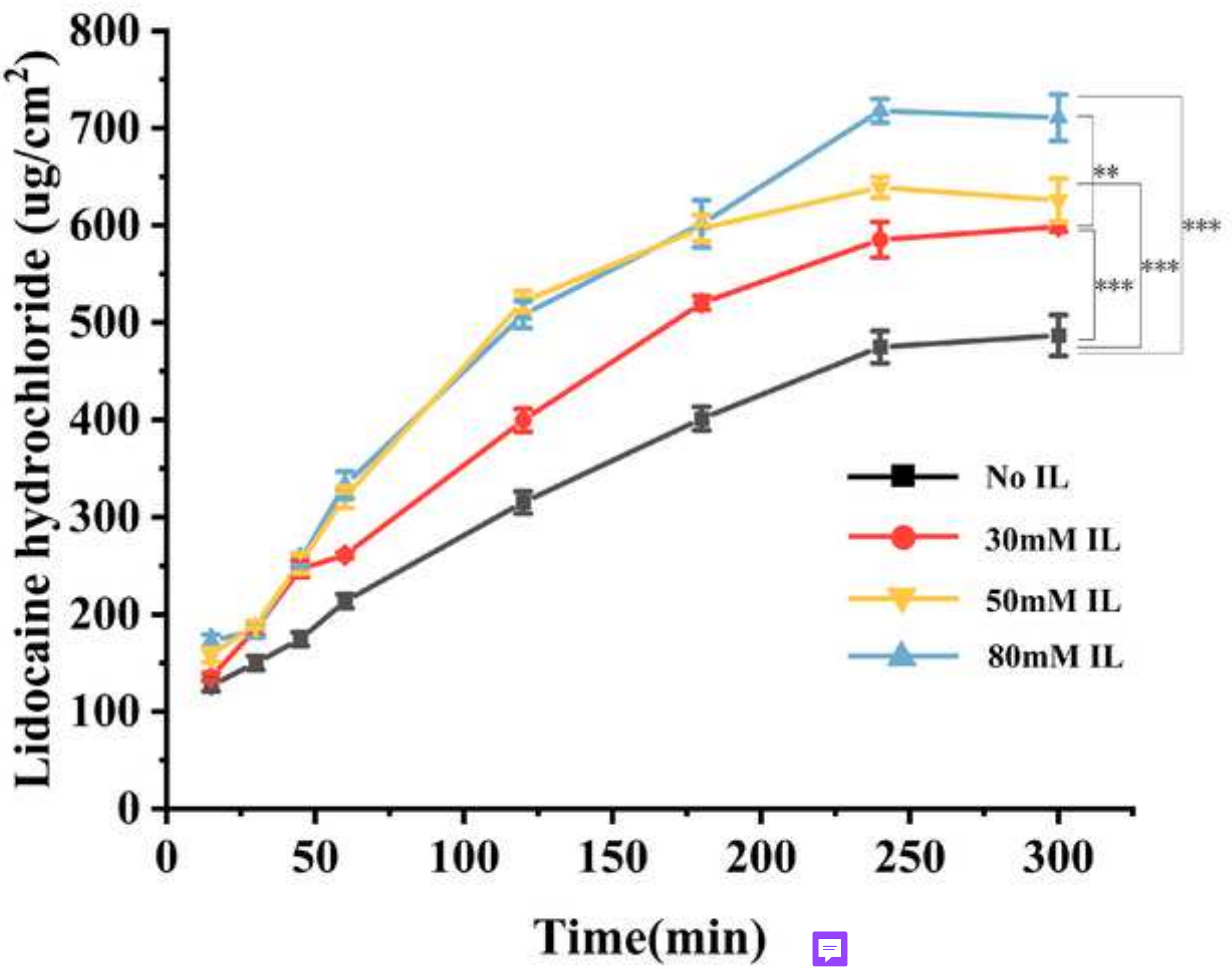

Click here to access/download
Supporting Information
Fig S1.tif

Click here to access/download
Supporting Information
Fig S2.tif

Revised Manuscript with Track Changes

Ionic-liquid-facilitated transdermal absorption of lidocaine hydrochloride

Xiaoting Ma[a], Yangzhe Zhou[b,*], Ke Jiang[a], Yuqi Wen[a,*]

[a] Department of Traditional Chinese Medicine, Xiangya Hospital, Central South University, Changsha 410000, China
[b] Department of Plastic surgery, Xiangya Hospital, Central South University, Changsha 410000, China

[*] Corresponding authors Email: zo7858667@gmail.com (Y. Zhou)
[*] Corresponding authors Email: wenyuqi1221@163.com (Y. Wen)

Abstract

The increasing worldwide need for analgesics, along with patients' aversion to injectable anesthetics and limited compliance, highlights the necessity for noninvasive local anesthesia. However, the skin barrier poses a significant obstacle to the efficient transdermal administration of anesthetics. In this study, we synthesized a new ionic liquid (IL), tetradecylphosphonium hexanoate, by mixing sodium hexanoate and CYPHOS 101 at equimolar ratios. We systematically evaluated the capacity of this IL to improve the transdermal transfer of lidocaine hydrochloride through in vitro skin permeation tests and cytotoxicity investigations in HaCaT cells. The IL significantly enhanced the percutaneous absorption of lidocaine in a concentration-dependent manner. At 80 mM, the permeation efficiency increased by approximately 1.5 times compared with the control group, resulting in a significantly reduced onset time for topical anesthesia. While IL showed minimal cytotoxicity at low concentrations, higher concentrations were linked to increased cytotoxicity. ILs can effectively enhance the permeation of transdermal lidocaine. The ease of synthesizing ILs, their adjustable properties, and good compatibility make them promising for creating advanced topical anesthetics and broadening transdermal drug delivery applications.

Keywords: lidocaine hydrochloride, ionic liquid, transdermal absorption, , analgesic delivery

Formatted: Font: 18 pt

1. Introduction

Formatted: Font: 18 pt

Pain can result from various diseases and injuries, significantly reducing quality

of life and imposing substantial economic and social burdens (1-6). For instance, the global prevalence of low back pain was estimated at 619 million in 2020, projected to reach 843 million by 2050, consistently ranking it as the primary cause of disability burden (7). Lidocaine hydrochloride is a commonly used amide-type local anesthetic in clinical practice and serves as a Class Ib antiarrhythmic agent for treating ventricular arrhythmias. Its primary mechanism involves blocking voltage-dependent sodium channels, thereby inhibiting the generation and conduction of action potentials in nerves or cardiac muscles (8). In anesthesiology, lidocaine provides a rapid onset, good penetration, and effective surface anesthesia for various clinical scenarios, including infiltration, nerve block, and surface/mucosal anesthesia. Its potency and duration fall within a moderate range among commonly used local anesthetics, with favorable tissue diffusion owing to its physicochemical properties. Upon absorption into the bloodstream or intravenous administration, it exerts distinct dual excitatory and inhibitory effects on the central nervous system (8-13).

The most common clinical administration route for lidocaine hydrochloride is subcutaneous injection. However, this method is often accompanied by significant stinging and local discomfort, thereby reducing patient compliance (14-17). To overcome this limitation, researchers have developed various alternative delivery strategies, including ultrasound-mediated transdermal delivery ~~(18-20)~~, electroporation ~~(18-20)~~, gel formulations ~~(21-24)~~, microneedles ~~(25-28)~~, and transdermal delivery (18-31). Compared with other routes, transdermal delivery is considered advantageous owing to its noninvasiveness and avoidance of the first-pass

effect, significantly improving drug bioavailability and patient experience (12, 26, 32). However, the skin stratum corneum—the body's primary natural barrier—remains the main obstacle limiting effective transdermal drug absorption (33).

In recent years, ionic liquids (ILs) have attracted significant attention due to their distinct physicochemical properties and remarkable capacity to enhance skin permeability(34-39). Comprising specific ratios of organic cations to organic/inorganic anions, IL typically possess melting points below 100 °C and remain liquid at room temperature, displaying outstanding solubility, thermal stability, and adjustability (34-39). These attributes hold great promise for drug delivery applications. Mandal et al. significantly enhanced the transdermal delivery and therapeutic effectiveness of NFKBIZ siRNA by combining it with IL for psoriasis treatment (40). Banerjee et al. utilized IL to enhance the oral absorption efficiency of insulin, presenting new opportunities for diabetes management (41). Therefore, ILs are not only innovative transdermal permeation enhancers but also poised to serve as a vital platform for delivering complex drugs and biologics in the future.

In this study, we aimed to utilize a novel IL, tetradecylphosphonium hexanoate, synthesized from CYPHOS 101 and sodium hexanoate, to enhance the transdermal absorption of lidocaine hydrochloride. We hypothesized that this IL would disrupt the stratum corneum barrier and increase drug permeation while maintaining acceptable biocompatibility. To test this hypothesis, we systematically evaluated the permeation-enhancing efficacy through in vitro skin permeation assays and assessed preliminary safety via cytotoxicity studies in HaCaT keratinocytes.

Formatted: Indent: First line: 2 ch, Line spacing: Double

In this study, we effectively utilized ILs to enhance the transdermal absorption of lidocaine hydrochloride. The specific IL employed was tetraalkylphosphonic acid hexanoate, synthesized by combining trihexyl tetradecylphosphonium chloride (CYPHOS 101) with sodium hexanoate in a 1:1 ratio . Skin permeation tests conducted in vitro confirmed the enhanced transdermal absorption of lidocaine hydrochloride facilitated by the IL. At an IL concentration of 80 mM, the transdermal absorption efficiency of lidocaine hydrochloride increased by 1.5 times, significantly reducing the wait time for epidermal anesthesia and addressing medical resource limitations. Furthermore, we evaluated the cytotoxicity of ILs by exposing immortalized human keratinocytes (HACAT) to varying IL concentrations, revealing minimal cytotoxic effects at low concentrations.

2. Materials and mMethods

Formatted: Font: 18 pt

2.1 Materials

Formatted: Font: 16 pt

Sodium hexanoate (99% purity) was acquired from Aladdin. Trihexyltetradecylphosphonium (tetraalkylphosphonium) chloride (CYPHOS 101, purity ≥ 97%), lidocaine hydrochloride (purity: 99%), and silver nitrate solution were procured from Macklin. Phosphate buffer for cell culture was purchased from Procell. Dimethyl sulfoxide (DMSO) and MTT for cell proliferation assays were sourced from SolarBio. HAaCaAT cells were obtained from Xiangya Hospital of Central South University. All reagents were of analytical grade and used without additional purification.

2.2 Instruments

Transdermal testing utilized a Franz diffusion cell (TK-12D, Shanghai Kaikai). Characterization was performed using nuclear magnetic resonance (NMR) on a Bruker 400 MHz (SwitzerlandGermany). Ultraviolet–visible (UV–vis) absorption spectra were obtained with a UV-2450 spectrophotometer (SHIMADZU, Japan). The Elx800 microplate reader (BioTek, U.S) was utilized.

Formatted: Font: 16 pt

2.3 Animal preparation

Male SD rats (weight: 180–220 g, age: 7–8 weeks) were provided by the Xiangya School of Medicine, Central South University(ethics audit number: CSU-2022-0170). All animals were housed under standard conditions (room temperature, 12-h light/dark cycle) with free access to a standard pellet diet and water. After a one-week acclimatization period, the rats were deeply anesthetized by an intraperitoneal injection of pentobarbital sodium (50 mg/kg). Euthanasia was subsequently performed via an overdose of the same anesthetic, and death was confirmed by the absence of breathing and cardiac arrest. The abdominal hair was then shaved, and the full-thickness abdominal skin was surgically excised. All subcutaneous fat and connective tissue were carefully removed.The SD rats (weight: 180-220g, age: 7-8 weeks) utilized in this investigation were provided by the Xiangya School of Medicine, Central South University (ethics audit number: CSU-2022-0170). The rats were kept at room temperature, under a 12-h light/dark cycle, and were given a standard pellet diet along with water ad libitum. Following a one-week acclimatization period, the

Formatted: Font: 16 pt

rats were anesthetized using pentobarbital sodium. Subsequently, their abdominal hair was removed with a shaver, and the abdominal skin was meticulously dissected, ensuring thorough cleaning of the subcutaneous fat layer and fascia. The excised skin samples were stored at −20 °C in a freezer and used within two weeks to preserve the integrity of the skin barrier for consistent in vitro permeation studies.The skin samples were then stored at −20 °C in a freezer and utilized within a two-week timeframe. The experiment was conducted in compliance with the Code of Ethics of the World Medical Association and approved by the Experimental Animal Welfare and Ethics Committee of Xiangya School of Medicine, Central South University.

2.4 In vitro skin permeation assays

Formatted: Font: 16 pt

The IL at concentrations of 30, 50, and 80 mM was combined with lidocaine hydrochloride and assessed for transdermal absorption efficiency. The transdermal testing apparatus, as illustrated in Fig. 1, consisted of a donor chamber positioned at the top, SD mouse skin treated in the middle (effective diffusion area: 3.14 cm^2), and an acceptor chamber at the bottom with a capacity of 8 mL.

First, a small magnet was positioned in the acceptor chamber, with skin placed between the acceptor and donor chambers. The stratum corneum (SC) of the skin was elevated, and rat skin was slightly stretched to minimize wrinkles. Subsequently, 8 mL of phosphate-buffered saline (PBS) was added to the acceptor chamber. Particular attention was paid to the discharge of subcutaneous air bubbles; otherwise, the experimental results would have been affected. Then, 3 mL of IL/lidocaine hydrochloride solution with different IL concentrations was added to each donor

~~chamber. The donor chamber was sealed with a film containing small holes to reduce evaporation. At regular intervals, 200 µmL of the sample was removed from the sampling port and supplemented with 200 µmL of PBS to keep the volume of the acceptor chamber constant. The chamber is placed in a magnetic box at a constant temperature of 37 °C for experiments.~~

2.~~5~~4 Synthesis of the IL

First, sodium hexanoate (1.46 g, 0.011 mol) powder was dissolved in 15 mL of methanol. CYPHOS 101 (5.48 g, 0.011 mol) was then added to the solution and stirred for 15–20 min until fully mixed. Methanol was removed by rotary evaporation, and the IL was washed with deionized water until the washed water was chloride-free. IL was subsequently dried in a vacuum oven at 80 °C for 48 h. For the preparation of the lidocaine hydrochloride-IL, lidocaine hydrochloride powder was added to the IL to achieve a concentration of 1 mg/mL and thoroughly mixed.

2.~~6~~5 Detection of lidocaine hydrochloride

Lidocaine hydrochloride was prepared at concentrations of 2, 3, 4, 6, 8, and 10 µ~~m~~g/mL. Subsequently, 100 µ~~m~~L of each concentration was introduced into the cuvette for analysis with a UV spectrometer.

2.~~7~~6 Cytotoxicity test

To evaluate the cytotoxicity of IL, Ha~~A~~Ca~~A~~T cells were selected as the model due to their suitability in mimicking the human epidermal state (42). First, ~~100 mL of cell suspension containing~~ 1 × 10^4 cells in 100 µL of culture medium were seeded into each well of a 96-well plate and incubated overnight.~~per 100 mL was seeded into each~~

Formatted: Font: 16 pt
Formatted: Font: 16 pt
Formatted: Font: 16 pt
Formatted: Font: 16 pt
Formatted: Font: 16 pt
Formatted: Font: 16 pt

well of a 96-well plate and incubated overnight. Upon confirming cell adhesion, the culture medium was aspirated and replaced with varying concentrations of IL (30, 50, and 80 mM) for 5 h. Subsequently, the medium was exchanged with 100 μmL of Dulbecco's modified Eagle's medium (DMEM) and incubated for an additional 19 h (24 h in total). The control group was treated solely with DMEM throughout the experiment. Following this, 20 μmL of MTT solution (0.5 mg/mL) was added to each well and incubated for 4 h. After discarding the MTT solution, 150 μmL of DMSO was introduced, and the plate was agitated for 20 min on a balanced shaker. The absorbance was measured at 490 nm using a microplate reader.This assay evaluated in vitro cellular biocompatibility and was not designed to determine in vivo lethal dose (LD50) parameters.

2.7 In vitro skin permeation assays

The IL at concentrations of 30, 50, and 80 mM was combined with lidocaine hydrochloride (at a fixed concentration of 1 mg/mL) and assessed for transdermal absorption efficiency. The transdermal testing apparatus, as illustrated in Fig. 1, consisted of a donor chamber positioned at the top, SD mouse skin treated in the middle (effective diffusion area: 3.14 cm^2), and an acceptor chamber at the bottom with a capacity of 8 mL.

First, a small magnet was positioned in the acceptor chamber, with skin placed between the acceptor and donor chambers. The stratum corneum (SC) of the skin was elevated, and rat skin was slightly stretched to minimize wrinkles. Subsequently, 8 mL of phosphate-buffered saline (PBS) was added to the acceptor chamber. Particular

attention was paid to the discharge of subcutaneous air bubbles; otherwise, the experimental results would have been affected. Then, 3 mL of IL/lidocaine hydrochloride solution with different IL concentrations was added to each donor chamber. The donor chamber was sealed with a film containing small holes to reduce evaporation. At regular intervals, 200 μL of the sample was removed from the sampling port and supplemented with 200 μL of PBS to keep the volume of the acceptor chamber constant. The chamber is placed in a magnetic box at a constant temperature of 37 °C for experiments.

Fig 1.Schematic of the Franz diffusion cell setup for in vitro skin permeation studies. Schematic illustration of the Franz diffusion cell used for transdermal permeation experiments, showing donor, receptor, and skin membrane compartments.

2.8 Sstatistical analysis

Data are presented as mean ± standard deviation (SD) of at least three independent experiments. For skin permeation and cell viability studies, statistical significance was determined using one-way analysis of variance (ANOVA) followed by Tukey's post hoc test for multiple comparisons. A p-value of less than 0.05 was considered statistically significant. All analyses were performed using GraphPad Prism software.

3. Results ~~and discussion~~

3.1 Characterization of the IL

The synthesized IL appeared as a clear, slightly yellow solution (Supporting

Formatted: Font: (Default) Times New Roman Regular, Bold

Formatted: Font: (Default) Times New Roman Regular, 12 pt, Bold

Formatted: Indent: First line: 0 ch

Formatted: Font: 12 pt

Formatted: Font: (Default) Times New Roman Regular, Bold

Formatted: Font: 16 pt

Formatted: Font: (Default) Times New Roman

Formatted: Font: 18 pt

Formatted: Font: 16 pt

Information, Fig. S1). The NMR spectrum of the IL was consistent with previous reports (Supporting Information, Fig. S2) (43): 1H NMR (400 MHz, Chloroform-d) δ 2.53 (s, 1H), 2.43 (ddt, J = 13.0, 9.6, 5.4 Hz, 2H), 1.63 (dd, J = 17.3, 5.4 Hz, 0H), 1.47 (t, J = 4.9 Hz, 0H), 1.45–1.25 (m, 7H), 0.87 (d, J = 3.3 Hz, 0H); and 13C NMR (101 MHz, Chloroform-d) δ 179.22, 38.40, 32.09, 31.76, 30.94, 30.70, 30.55, 30.37, 30.22, 29.48, 29.38, 29.19, 28.83, 26.47, 22.55, 22.52, 22.27, 22.20, 21.68, 19.06, 18.59, 13.99, 13.96, and 13.77.

3.2 Detection of lidocaine hydrochloride

As shown in Fig. 2, the absorbance intensity of the lidocaine hydrochloride solution at approximately 196 nm increased with increasing lidocaine hydrochloride concentrations within the 2–10 μ~~m~~g/mL range, exhibiting a correlation coefficient of 0.999. These absorbance readings were subsequently employed in a subsequent experiment to evaluate the transdermal administration of lidocaine hydrochloride.

Fig 2.UV–Vis calibration of lidocaine hydrochloride. UV absorption spectra of lidocaine hydrochloride at different concentrations (a–f: 2, 3, 4, 6, 8, and 10 μg mL^{-1}) and calibration curve showing the linear relationship between absorbance at 196 nm and lidocaine concentration

3.3 HaCaT cell viability evaluation of the IL

The cytotoxicity of the IL towards HaCaT keratinocytes was evaluated, and the results are presented in Fig. 3. The cell viability relative to the untreated control was 89.3 ± 1.9% for the 30 mM IL group and 88.9 ± 3.1% for the 50 mM IL group. There

Formatted: Font: 16 pt

Formatted: Font: (Default) Times New Roman Bold

Formatted: Font: (Default) Times New Roman Bold, 12 pt

Formatted: Font: (Default) Times New Roman Regular, 12 pt

Formatted: Font: (Default) Times New Roman Regular, 12 pt, Not Bold

Formatted: Line spacing: Double

Formatted: Indent: First line: 0 ch

Formatted: Indent: First line: 2 ch

was no statistically significant difference between these two groups ($p > 0.05$, Tukey's test). In contrast, exposure to 80 mM IL significantly reduced cell viability to 41.2 ± 0.8% of the control level ($p < 0.001$ versus the control, 30 mM, and 50 mM groups; one-way ANOVA with Tukey's post hoc test).

Fig 3. Cytotoxicity of ionic liquids in HaCaT cells. Viability of HaCaT cells treated with different concentrations of ionic liquids (ILs). After 5 h of IL exposure, the medium was replaced with fresh DMEM and cells were incubated for an additional 19 h (24 h total). Cell viability was assessed using the MTT assay. Data are presented as mean ± SD (n = 5). ***$p < 0.001$ versus the control group (one-way ANOVA with Tukey's post hoc test).

3.34 In vitro skin permeation assays

As shown in Fig. 34, the cumulative permeation of lidocaine hydrochloride increased in a time and IL concentration-dependent manner. The permeation approached a plateau after approximately 4 hours. At the 5-hour time point, the cumulative amount permeated in the presence of 80 mM IL was approximately 1.5-fold higher than that in the control group (without IL), and this difference was statistically significant ($p < 0.05$). Both 50 mM and 80 mM IL groups showed significant enhancement compared to the control ($p < 0.05$) from the 3-hour time point onward, with no significant difference between these two IL groups observed within the first 3 hours.As shown in Fig. 3, the transdermally delivered lidocaine hydrochloride increased gradually over time, reaching saturation at 4 h. With the gradual increase in IL concentration, the amount of lidocaine hydrochloride delivered

Formatted: Font: (Default) Times New Roman, Not Italic

Formatted: Font: (Default) Times New Roman, Not Italic

Formatted: Font: (Default) Times New Roman Regular

Formatted: Caption

Formatted: Font: (Default) Times New Roman Regular, 12 pt

Formatted: Font: (Default) Times New Roman Regular, 12 pt, Not Bold

Formatted: Font: Times New Roman, 12 pt, Not Bold

Formatted: Font: (Default) Times New Roman Regular, 12 pt, Not Bold

Formatted: Font: (Default) Times New Roman Regular

Formatted: Font: 16 pt

Formatted: Font: 16 pt

Formatted: Font: (Default) Times New Roman, Not Italic

Formatted: Font: (Default) Times New Roman, Not Italic

also gradually increased over the same period. Our data showed that when the concentration of IL was 80 mM, the amount of lidocaine hydrochloride delivered at the 5th hour was nearly 1.5 times that of the blank control group that did not receive IL. Moreover, for the 50 and 80 mM IL groups, the delivery rates of lidocaine hydrochloride were essentially the same before the first 3 h, but the 80 mM group promoted penetration more efficiently after 3 h. However, in clinical situations, the waiting time of patients for epidermal local anesthesia is usually less than 1 h. Therefore, an IL concentration of 50 mM is optimal for practical applications.

3.4 HaACaAT cell viability evaluation of the IL

Formatted: Font: 16 pt

The cytotoxicity of the IL towards HaCaT keratinocytes was evaluated, and the results are presented in Fig. 4. The cell viability relative to the untreated control was 89.3 ± 1.9% for the 30 mM IL group and 88.9 ± 3.1% for the 50 mM IL group. There was no statistically significant difference between these two groups ($p > 0.05$, Tukey's test). In contrast, exposure to 80 mM IL significantly reduced cell viability to 41.2 ± 0.8% of the control level ($p < 0.001$ versus the control, 30 mM, and 50 mM groups; one-way ANOVA with Tukey's post hoc test).The biocompatibility of the IL was assessed through a cytotoxicity assay. As shown in Fig. 4, when HACAT cells were exposed to 30 and 50 mM IL, cell viability was reduced by 11% and 12%, respectively, compared with the control group. This demonstrates the low toxicity of the IL towards HACAT cells at these concentrations, suggesting potential application in future human trials. In contrast, at an IL concentration of 80 mM, HACAT cell viability significantly decreased to 41% of the control group, indicating pronounced

cytotoxic effects. The optimal IL concentration for clinical use, as previously discussed, is 50 mM, where the minimal cytotoxicity of the IL supports its broad clinical applications.

Fig 4.Effect of ionic liquid concentration on lidocaine permeation. Enhanced transdermal absorption efficiency of lidocaine hydrochloride in the presence of different concentrations of ionic liquids (ILs). Data points represent the mean ± SD (n = 3). Asterisks indicate statistically significant differences compared to the control group at the corresponding time point (** $p < 0.01$, *** $p < 0.001$, one-way ANOVA with Tukey's post hoc test).

4. Discussion

This study demonstrates that the synthesized IL significantly enhanced the transdermal permeation of lidocaine hydrochloride in a concentration-dependent manner. The 1.5-fold increase in cumulative permeation achieved with 80 mM IL suggests a strong potential to shorten the onset time of topical anesthesia, addressing a key clinical need for rapid-acting, non-invasive analgesics. Critically, we identified 50 mM as an optimal concentration for further development. At this level, the IL provided substantial permeation enhancement while maintaining high viability in HaCaT cells, indicating a favorable balance between efficacy and biocompatibility. This concentration is particularly relevant given that the acceptable waiting time for topical anesthesia in clinical practice is typically under one hour, a duration within which the 50 mM formulation demonstrates effective delivery. However, these promising in vitro findings are subject to limitations. Future work must include in

Formatted: Font: (Default) Times New Roman Bold

Formatted: Font: (Default) Times New Roman Bold

Formatted: Caption, Indent: First line: 2 ch

Formatted: Font: (Default) Times New Roman Bold, 12 pt

Formatted: Font: (Default) Times New Roman Regular, 12 pt, Not Bold

Formatted: Font: Times New Roman, 12 pt, Not Bold

Formatted: Font: (Default) Times New Roman Regular, 12 pt, Not Bold

Formatted: Font: Times New Roman, 12 pt, Not Bold

Formatted: Font: (Default) Times New Roman Regular, 12 pt, Not Bold

Formatted: Font: Not Bold

Formatted: Indent: First line: 0 ch, Line spacing: single

Formatted: Font: 18 pt

Formatted: Indent: First line: 2 ch, No bullets or numbering

vivo models to validate the anesthetic efficacy and systemic safety, employ more complex skin models (e.g., reconstructed human epidermis) for comprehensive safety assessment, and investigate the formulation's long-term stability. Despite these necessary next steps, the current results establish a solid foundation for the development of advanced IL-facilitated transdermal drug delivery systems.

Formatted: Indent: First line: 0 ch, Line spacing: single

4.5. Conclusions

Formatted: Font: 18 pt

In this study, a novel tetraalkylphosphonium hexanoate IL was successfully synthesized. In vitro evaluations demonstrated that this IL significantly enhanced the transdermal permeation of lidocaine hydrochloride in a concentration-dependent manner, with an approximately 1.5-fold increase observed at 80 mM compared to the control. Importantly, at the effective concentration of 50 mM, the IL exhibited minimal cytotoxicity against HaCaT keratinocytes. These findings collectively support the potential of this IL as an effective and biocompatible permeation enhancer for topical lidocaine delivery.Future studies should focus on the formulation development of a practical transdermal delivery system and the preclinical evaluation of lidocaine bioavailability in vivo.

Formatted: Indent: First line: 2 ch, Line spacing: Double

This study successfully synthesized a tetraalkylphosphine hexanoate IL by combining sodium hexanoate with CYPHOS 101 in an equimolar ratio. The impact of this IL on the transdermal absorption of lidocaine hydrochloride was systematically evaluated. The results demonstrated a significant enhancement in the transdermal absorption of lidocaine hydrochloride with increasing IL concentration. At 80 mM,

the transdermal absorption efficiency was nearly 1.5 times higher than that of the control group. This finding suggests that this strategy may shorten the time required for local anesthesia, thereby optimizing clinical procedures and alleviating healthcare resource constraints. However, it is crucial to acknowledge that IL cytotoxicity escalates with rising IL concentration. Subsequent investigations should prioritize mitigating IL cytotoxicity and improving biocompatibility to advance its clinical utility in local anesthesia and transdermal drug delivery.

Acknowledgements

Formatted: Font: 18 pt

The authors thank colleagues for their technical assistance and valuable discussions.

Formatted: Indent: First line: 2 ch, Line spacing: Double

This work was supported by the National Science Foundation of China (8207104259), the Hunan Provincial Science and Technology Plan Project (No. 2019TP1001), and the Innovation Driven Project of Central South University (2020CX002); we also thank all team members for their assistance with the experiment and data analysis.

Author contributions:

Formatted: Font: 18 pt

Yuqi Wen, Xiaoting Ma, Yangzhe Zhou: Conception and design of the study; Acquisition of data; Analysis and interpretation of data; Provision of reagents, materials, analysis tools, or data; Drafting the manuscript. Ke Jiang: Conception and design of the study; Analysis and interpretation of data.

Competing interests

Formatted: Font: 18 pt

The authors declare no competing interests.

~~Funding sources~~

Formatted: Font: 18 pt

~~This work was supported by the National Science Foundation of China (8207104259), the Hunan Provincial Science and Technology Plan Project, China (No. 2019TP1001), and the Innovation Driven Project of Central South University (2020CX002).~~

Formatted: Indent: First line: 2 ch

Data availability

Formatted: Font: 18 pt

All data generated or analysed during this study are included in this published article (and its Supplementary Information files).

References

Formatted: Font: 18 pt

1. Cagle JG, Carr DC, Hong S, Zimmerman S. Financial burden among US households affected by cancer at the end of life. Psychooncology. 2016;25(8):919-26.
2. Fitzcharles MA, Cohen SP, Clauw DJ, Littlejohn G, Usui C, Häuser W. Nociplastic pain: towards an understanding of prevalent pain conditions. Lancet. 2021;397(10289):2098-110.
3. Jiang C, Wang H, Wang Q, Luo Y, Sidlow R, Han X. Prevalence of Chronic Pain and High-Impact Chronic Pain in Cancer Survivors in the United States. JAMA Oncol. 2019;5(8):1224-6.
4. Paice JA. Cancer pain management and the opioid crisis in America: How to preserve hard-earned gains in improving the quality of cancer pain management. Cancer. 2018;124(12):2491-7.
5. Shkodra M, Brunelli C, Zecca E, Formaglio F, Bracchi P, Lo Dico S, et al. Neuropathic pain: clinical classification and assessment in patients with pain due to cancer. Pain. 2021;162(3):866-74.
6. van den Beuken-van Everdingen MH, Hochstenbach LM, Joosten EA, Tjan-Heijnen VC, Janssen DJ. Update on Prevalence of Pain in Patients With Cancer: Systematic Review and Meta-Analysis. J Pain Symptom Manage. 2016;51(6):1070-90.e9.
7. Global, regional, and national burden of low back pain, 1990-2020, its attributable risk

factors, and projections to 2050: a systematic analysis of the Global Burden of Disease Study 2021. Lancet Rheumatol. 2023;5(6):e316-e29.

8. Taylor A, McLeod G. Basic pharmacology of local anaesthetics. BJA Educ. 2020;20(2):34-41.

9. Bahar E, Yoon H. Lidocaine: A Local Anesthetic, Its Adverse Effects and Management. Medicina (Kaunas). 2021;57(8).

10. Beaussier M, Delbos A, Maurice-Szamburski A, Ecoffey C, Mercadal L. Perioperative Use of Intravenous Lidocaine. Drugs. 2018;78(12):1229-46.

11. de Carvalho JF, Skare TL. Lidocaine in fibromyalgia: A systematic review. World J Psychiatry. 2022;12(4):615-22.

12. Wang Y, Su W, Li Q, Li C, Wang H, Li Y, et al. Preparation and evaluation of lidocaine hydrochloride-loaded TAT-conjugated polymeric liposomes for transdermal delivery. Int J Pharm. 2013;441(1-2):748-56.

13. Zhang D, Ye D, Jing P, Tan X, Qiu L, Li T, et al. Design, optimization and evaluation of co-surfactant free microemulsion-based hydrogel with low surfactant for enhanced transdermal delivery of lidocaine. Int J Pharm. 2020;586:119415.

14. Chu CC, Wu SZ, Su WL, Shieh JP, Kao CH, Ho ST, et al. Subcutaneous injection of inhaled anesthetics produces cutaneous analgesia. Can J Anaesth. 2008;55(5):290-4.

15. Ellis BD, Kosmorsky GS. Referred ocular pain relieved by suboccipital injection. Headache. 1995;35(2):101-3.

16. Klein JA, Jeske DR. Estimated Maximal Safe Dosages of Tumescent Lidocaine. Anesth Analg. 2016;122(5):1350-9.

17. Levin M. Nerve blocks in the treatment of headache. Neurotherapeutics. 2010;7(2):197-203.

18. Batheja P, Thakur R, Michniak B. Transdermal iontophoresis. Expert Opin Drug Deliv. 2006;3(1):127-38.

19. Ito Y, Ohta J, Imada K, Akamatsu S, Tsuchida N, Inoue G, et al. Dissolving microneedles to obtain rapid local anesthetic effect of lidocaine at skin tissue. J Drug Target. 2013;21(8):770-5.

20. Kathuria H, Li H, Pan J, Lim SH, Kochhar JS, Wu C, et al. Large Size Microneedle Patch to Deliver Lidocaine through Skin. Pharm Res. 2016;33(11):2653-67.

21. Kochhar JS, Lim WX, Zou S, Foo WY, Pan J, Kang L. Microneedle integrated transdermal patch for fast onset and sustained delivery of lidocaine. Mol Pharm. 2013;10(11):4272-80.

22. Li Y, Zhao E, Li L, Bai L, Zhang W. Facile design of lidocaine-loaded polymeric hydrogel to persuade effects of local anesthesia drug delivery system: complete in vitro and in vivo toxicity analyses. Drug Deliv. 2021;28(1):1080-92.

23. Liu DZ, Sheu MT, Chen CH, Yang YR, Ho HO. Release characteristics of lidocaine from local implant of polyanionic and polycationic hydrogels. J Control Release. 2007;118(3):333-9.

24. Long J, Etxeberria AE, Nand AV, Bunt CR, Ray S, Seyfoddin A. A 3D printed chitosan-pectin hydrogel wound dressing for lidocaine hydrochloride delivery. Mater Sci Eng C Mater Biol Appl. 2019;104:109873.

25. Nayak A, Babla H, Han T, Das DB. Lidocaine carboxymethylcellulose with gelatine co-polymer hydrogel delivery by combined microneedle and ultrasound. Drug Deliv. 2016;23(2):658-69.

26. Prausnitz MR, Langer R. Transdermal drug delivery. Nat Biotechnol. 2008;26(11):1261-8.

27. Rasool A, Ata S, Islam A, Rizwan M, Azeem MK, Mehmood A, et al. Kinetics and controlled

release of lidocaine from novel carrageenan and alginate-based blend hydrogels. Int J Biol Macromol. 2020;147:67-78.
28. Sammeta SM, Vaka SR, Murthy SN. Transdermal drug delivery enhanced by low voltage electropulsation (LVE). Pharm Dev Technol. 2009;14(2):159-64.
29. Soto F, Jeerapan I, Silva-López C, Lopez-Ramirez MA, Chai I, Xiaolong L, et al. Noninvasive Transdermal Delivery System of Lidocaine Using an Acoustic Droplet-Vaporization Based Wearable Patch. Small. 2018;14(49):e1803266.
30. Tachibana K, Tachibana S. Use of ultrasound to enhance the local anesthetic effect of topically applied aqueous lidocaine. Anesthesiology. 1993;78(6):1091-6.
31. Wallace MS, Ridgeway B, Jun E, Schulteis G, Rabussay D, Zhang L. Topical delivery of lidocaine in healthy volunteers by electroporation, electroincorporation, or iontophoresis: an evaluation of skin anesthesia. Reg Anesth Pain Med. 2001;26(3):229-38.
32. Yuan JS, Ansari M, Samaan M, Acosta EJ. Linker-based lecithin microemulsions for transdermal delivery of lidocaine. Int J Pharm. 2008;349(1-2):130-43.
33. Ahad A, Aqil M, Kohli K, Chaudhary H, Sultana Y, Mujeeb M, et al. Chemical penetration enhancers: a patent review. Expert Opin Ther Pat. 2009;19(7):969-88.
34. Curreri AM, Mitragotri S, Tanner EEL. Recent Advances in Ionic Liquids in Biomedicine. Adv Sci (Weinh). 2021;8(17):e2004819.
35. Egorova KS, Gordeev EG, Ananikov VP. Biological Activity of Ionic Liquids and Their Application in Pharmaceutics and Medicine. Chem Rev. 2017;117(10):7132-89.
36. Lei Z, Chen B, Koo YM, MacFarlane DR. Introduction: Ionic Liquids. Chem Rev. 2017;117(10):6633-5.
37. Qi QM, Mitragotri S. Mechanistic study of transdermal delivery of macromolecules assisted by ionic liquids. J Control Release. 2019;311-312:162-9.
38. Tanner EEL, Curreri AM, Balkaran JPR, Selig-Wober NC, Yang AB, Kendig C, et al. Design Principles of Ionic Liquids for Transdermal Drug Delivery. Adv Mater. 2019;31(27):e1901103.
39. Zakrewsky M, Lovejoy KS, Kern TL, Miller TE, Le V, Nagy A, et al. Ionic liquids as a class of materials for transdermal delivery and pathogen neutralization. Proc Natl Acad Sci U S A. 2014;111(37):13313-8.
40. Mandal A, Kumbhojkar N, Reilly C, Dharamdasani V, Ukidve A, Ingber DE, et al. Treatment of psoriasis with NFKBIZ siRNA using topical ionic liquid formulations. Sci Adv. 2020;6(30):eabb6049.
41. Banerjee A, Ibsen K, Brown T, Chen R, Agatemor C, Mitragotri S. Ionic liquids for oral insulin delivery. Proc Natl Acad Sci U S A. 2018;115(28):7296-301.
42. Cui D, Bi J, Zhang ZN, Li MY, Qin YS, Xiang P, et al. Organophosphorus flame retardant TDCPP-induced cytotoxicity and associated mechanisms in normal human skin keratinocytes. Sci Total Environ. 2020;726:138526.
43. Oster K, Goodrich P, Jacquemin J, Hardacre C, Ribeiro APC, Elsinawi A. A new insight into pure and water-saturated quaternary phosphonium-based carboxylate ionic liquids: Density, heat capacity, ionic conductivity, thermogravimetric analysis, thermal conductivity and viscosity. The Journal of Chemical Thermodynamics. 2018;121:97-111.

Supporting information

S1 Fig Appearance of the synthesized ionic liquid (IL) Photograph showing the physical state of the synthesized ionic liquid. The IL presents as a clear and slightly yellow solution.

S2 Fig Nuclear Magnetic Resonance (NMR) spectrum of the synthesized ionic liquid (IL) NMR spectrum of the target synthesized ionic liquid. The characteristic resonance peaks observed in the spectrum are consistent with those reported in previous studies, which confirms the successful synthesis and structural integrity of the IL.

Formatted: Font: (Default) Times New Roman Bold, 12 pt

Formatted: Line spacing: Double

Formatted: Font: (Default) Times New Roman Regular, 12 pt, Not Bold

Formatted: Font: (Default) Times New Roman Bold, 12 pt

Formatted: Font: (Default) Times New Roman Regular, 12 pt, Not Bold

Response to Reviewers

Manuscript ID: PONE-D-25-63253
Title: Ionic-liquid-facilitated transdermal absorption of lidocaine hydrochloride

Dear Academic Editor and Reviewers,

Thank you for the opportunity to revise our manuscript and for the constructive comments provided by the reviewers. We have carefully addressed all points raised and have made substantial revisions to improve the manuscript. Our point-by-point responses are detailed below. All changes in the manuscript have been highlighted using the “Track Changes” function.

Responses to Reviewer #1:

We sincerely thank Reviewer #1 for the thorough review and valuable suggestions.

1. Comment: The introduction contains a conclusion statement. “Skin permeation tests conducted in vitro confirmed…” should not appear in the introduction.
   Response: We agree and have revised the final paragraph of the Introduction section. The conclusive statements have been removed and replaced with a clear statement of the study’s aims and hypothesis.

2. Comment: Please consider adding a picture of the Franz diffusion cell setup.
   Response: A schematic diagram of the Franz diffusion cell setup is already provided as Fig. 1 in the original submission. We have now extended the title of this chart.

3. Comment: The sex of SD rats should be specified to exclude potential hormonal influences.
   Response: We have specified that male SD rats were used in the revised Section 2.3 (Animal preparation).

4. Comment: Please clarify why skin samples were stored at −20 °C and used within two weeks.
   Response: An explanation has been added to Section 2.3: skin samples were stored at −20 °C to preserve the integrity of the skin barrier and were used within two weeks to ensure experimental consistency in the in vitro permeation studies.

5. Comment: Does this study include in vivo results?
   Response: This study is limited to in vitro evaluations. We have acknowledged this as a key limitation in the new Section 4. Discussion (Limitations and future perspectives).

6. Comment: Please provide ED50/LD50 data from the toxicity test.
Response: The cytotoxicity assay was conducted on HaCaT cells to assess in vitro biocompatibility and was not designed to determine in vivo lethal doses (LD50). We have clarified this point in the Section 2.7 (Cytotoxicity test) and in the Discussion.

7. Comment: It is better to separate the Results and Discussion sections.
Response: As suggested, we have restructured the manuscript by separating the combined "Results and discussion" section into two distinct chapters: "3. Results" and "4. Discussion."

Responses to Reviewer #2:

We sincerely thank Reviewer #2 (Dr. Shashank Bhangde) for their thorough review, positive feedback, and constructive suggestions, which have significantly strengthened our manuscript.

1. Comment: Consider adding statistical analysis to the data where possible.
Response: We have performed comprehensive statistical analysis as suggested. A new Section 2.8 (Statistical analysis) has been added, describing the use of one-way ANOVA followed by Tukey's post hoc test. The error bars in Fig. 3 and Fig. 4 now represent the standard deviation (SD), and statistically significant differences are indicated with asterisks (**$p < 0.01$, ***$p < 0.001$). The results sections (3.3 and 3.4) now include explicit statements of statistical significance.

2. Comment: The specific term "analgesic drug delivery" was missing.
Response: We thank the reviewer for highlighting this important omission. To accurately frame the therapeutic focus of our work and enhance the manuscript's discoverability, we have added "analgesic drug delivery" as a keyword in the revised manuscript.

3. Comment: All citations appear at the end of the statement.
Response: We have reviewed and revised the manuscript to ensure all citations are placed at the end of their respective statements for consistency, including in the Introduction where general statements about lidocaine and ionic liquids were lacking support.

4. Comment: In current clinical setting, anesthesia is giving SC. You have only shown 1.5x increase in comparison to lidocaine given transdermally (which is not clinical standard). Rephrase this line.
Response: We thank the reviewer for this crucial clarification. We have rephrased the relevant line in the Conclusions section。

5. Comment: General suggestion to add future research directions.

Response: We appreciate this suggestion. As guided by the Editor, we have expanded the Conclusions to include a specific sentence on future work: "Future studies should focus on the formulation development of a practical transdermal delivery system and the preclinical evaluation of lidocaine bioavailability in vivo."

6. Comment: "I have a little flow issue. The flow should be: synthesis – detection – cytotoxicity – skin permeation."

Response: We agree with the reviewer's suggestion for a more logical narrative flow. In the revised manuscript, we have reorganized the "3. Results" section accordingly:

3.1 Characterization of the IL
3.2 Detection of lidocaine hydrochloride
3.3 HaCaT cell viability evaluation of the IL
3.4 In vitro skin permeation assays

We believe this new order—presenting the material, then its safety profile, followed by its functional efficacy—provides a clearer and more compelling argument. All in-text citations to figures and results have been updated to reflect this new structure.We have also reordered the corresponding sections in '2. Materials and methods' to maintain consistency with this improved narrative flow.

7. Comment:The issue of inconsistent units (ml, mg, μg)

Response: We thank the reviewer for identifying the unit errors. All instances of incorrect or ambiguous units have been identified and standardized throughout the manuscript to ensure accuracy and consistency.

Additional Revisions in Response to Journal Requirements:

We have also addressed all points raised by the Academic Editor to ensure full compliance with PLOS ONE policies.

1. Style Requirements: The manuscript has been adjusted as per the requirements and the structure has been ensured to comply with the guidelines.
2. ORCID iD: The corresponding author's ORCID iD is registered and validated in the submission system.
3. Animal Ethics Details: As requested, Section 2.3 has been expanded to include specific details on (1) the method of euthanasia (anesthetic overdose), (2) the method of anesthesia (intraperitoneal injection of pentobarbital sodium, 50 mg/kg), and (3) efforts to minimize suffering (all procedures performed under deep anesthesia).
4. NMR Data: Details regarding the NMR instrument (Bruker 400 MHz), magnetic field strength have been added to Section 2.2. The NMR spectrum is provided as Fig. S2 in the Supporting Information
5. & 6. & 7. Funding Information: As instructed, all funding-related text has been removed from the manuscript's Acknowledgements section. The funding statement provided in the submission system remains accurate. The role of the funders is stated

here, as requested for the cover letter: “The funders were involved in providing financial support for the experiment and in the decision to publish.”
8. & 9. Supporting Information: Captions for the Supporting Information files (Fig. S1 and Fig. S2) have been included at the end of the main manuscript file. The file types have been designated correctly in the submission system.

We believe the revised manuscript has been significantly improved and now fully meets the publication standards of PLOS ONE. We are grateful for the insightful comments and hope you find our revisions satisfactory.

Sincerely,

Yangzhe Zhou, Yuqi Wen
On behalf of all co-authors
