# Supplementary material for: Ionic-liquid-facilitated transdermal absorption of lidocaine hydrochloride
Source: PLoS One. 2026 Apr 3;21(4):e0344789. doi: 10.1371/journal.pone.0344789 (PMC13048389; doi:10.1371/journal.pone.0344789)
Supplement: Attachment — Submitted filename: Response to Reviewers.docx [file pone.0344789.s005.docx]

Response to Reviewers

Manuscript ID: PONE-D-25-63253

Title: Ionic-liquid-facilitated transdermal absorption of lidocaine hydrochloride

Dear Academic Editor and Reviewers,

Thank you for the opportunity to revise our manuscript and for the constructive comments provided by the reviewers. We have carefully addressed all points raised and have made substantial revisions to improve the manuscript. Our point-by-point responses are detailed below. All changes in the manuscript have been highlighted using the “Track Changes” function.

Responses to Reviewer #1:

We sincerely thank Reviewer #1 for the thorough review and valuable suggestions.

1. Comment: The introduction contains a conclusion statement. “Skin permeation tests conducted in vitro confirmed…” should not appear in the introduction.

Response: We agree and have revised the final paragraph of the Introduction section. The conclusive statements have been removed and replaced with a clear statement of the study’s aims and hypothesis.

2. Comment: Please consider adding a picture of the Franz diffusion cell setup.

Response: A schematic diagram of the Franz diffusion cell setup is already provided as Fig. 1 in the original submission. We have now extended the title of this chart.

3. Comment: The sex of SD rats should be specified to exclude potential hormonal influences.

Response: We have specified that male SD rats were used in the revised Section 2.3 (Animal preparation).

4. Comment: Please clarify why skin samples were stored at −20 °C and used within two weeks.

Response: An explanation has been added to Section 2.3: skin samples were stored at −20 °C to preserve the integrity of the skin barrier and were used within two weeks to ensure experimental consistency in the in vitro permeation studies.

5. Comment: Does this study include in vivo results?

Response: This study is limited to in vitro evaluations. We have acknowledged this as a key limitation in the new Section 4. Discussion (Limitations and future perspectives).

6. Comment: Please provide ED50/LD50 data from the toxicity test.

Response: The cytotoxicity assay was conducted on HaCaT cells to assess in vitro biocompatibility and was not designed to determine in vivo lethal doses (LD50). We have clarified this point in the Section 2.7 (Cytotoxicity test) and in the Discussion.

7. Comment: It is better to separate the Results and Discussion sections.

Response: As suggested, we have restructured the manuscript by separating the combined “Results and discussion” section into two distinct chapters: “3. Results” and “4. Discussion.”

Responses to Reviewer #2:

We sincerely thank Reviewer #2 (Dr. Shashank Bhangde) for their thorough review, positive feedback, and constructive suggestions, which have significantly strengthened our manuscript.

1. Comment: Consider adding statistical analysis to the data where possible.

Response: We have performed comprehensive statistical analysis as suggested. A new Section 2.8 (Statistical analysis) has been added, describing the use of one-way ANOVA followed by Tukey’s post hoc test. The error bars in Fig. 3 and Fig. 4 now represent the standard deviation (SD), and statistically significant differences are indicated with asterisks (**p < 0.01, ***p < 0.001). The results sections (3.3 and 3.4) now include explicit statements of statistical significance.

2. Comment: The specific term “analgesic drug delivery” was missing.

Response: We thank the reviewer for highlighting this important omission. To accurately frame the therapeutic focus of our work and enhance the manuscript’s discoverability, we have added “analgesic drug delivery” as a keyword in the revised manuscript.

3. Comment: All citations appear at the end of the statement.

Response: We have reviewed and revised the manuscript to ensure all citations are placed at the end of their respective statements for consistency, including in the Introduction where general statements about lidocaine and ionic liquids were lacking support.

4. Comment: In current clinical setting, anesthesia is giving SC. You have only shown 1.5x increase in comparison to lidocaine given transdermally (which is not clinical standard). Rephrase this line.

Response: We thank the reviewer for this crucial clarification. We have rephrased the relevant line in the Conclusions section。

5. Comment: General suggestion to add future research directions.

Response: We appreciate this suggestion. As guided by the Editor, we have expanded the Conclusions to include a specific sentence on future work: “Future studies should focus on the formulation development of a practical transdermal delivery system and the preclinical evaluation of lidocaine bioavailability in vivo.”

6. Comment: “I have a little flow issue. The flow should be: synthesis – detection – cytotoxicity – skin permeation.”

Response: We agree with the reviewer’s suggestion for a more logical narrative flow. In the revised manuscript, we have reorganized the “3. Results” section accordingly:

3.1 Characterization of the IL

3.2 Detection of lidocaine hydrochloride

3.3 HaCaT cell viability evaluation of the IL

3.4 In vitro skin permeation assays

We believe this new order—presenting the material, then its safety profile, followed by its functional efficacy—provides a clearer and more compelling argument. All in-text citations to figures and results have been updated to reflect this new structure.We have also reordered the corresponding sections in ‘2. Materials and methods’ to maintain consistency with this improved narrative flow.

7. Comment:The issue of inconsistent units (ml, mg, µg)

Response: We thank the reviewer for identifying the unit errors. All instances of incorrect or ambiguous units have been identified and standardized throughout the manuscript to ensure accuracy and consistency.

Additional Revisions in Response to Journal Requirements:

We have also addressed all points raised by the Academic Editor to ensure full compliance with PLOS ONE policies.

1. Style Requirements: The manuscript has been adjusted as per the requirements and the structure has been ensured to comply with the guidelines.

2. ORCID iD: The corresponding author’s ORCID iD is registered and validated in the submission system.

3. Animal Ethics Details: As requested, Section 2.3 has been expanded to include specific details on (1) the method of euthanasia (anesthetic overdose), (2) the method of anesthesia (intraperitoneal injection of pentobarbital sodium, 50 mg/kg), and (3) efforts to minimize suffering (all procedures performed under deep anesthesia).

4. NMR Data: Details regarding the NMR instrument (Bruker 400 MHz), magnetic field strength have been added to Section 2.2. The NMR spectrum is provided as Fig. S2 in the Supporting Information

5. & 6. & 7. Funding Information: As instructed, all funding-related text has been removed from the manuscript’s Acknowledgements section. The funding statement provided in the submission system remains accurate. The role of the funders is stated here, as requested for the cover letter: “The funders were involved in providing financial support for the experiment and in the decision to publish.”

8. & 9. Supporting Information: Captions for the Supporting Information files (Fig. S1 and Fig. S2) have been included at the end of the main manuscript file. The file types have been designated correctly in the submission system.

We believe the revised manuscript has been significantly improved and now fully meets the publication standards of PLOS ONE. We are grateful for the insightful comments and hope you find our revisions satisfactory.

Sincerely,

Yangzhe Zhou, Yuqi Wen

On behalf of all co-authors
