# Supplementary material for: Ionic-liquid-facilitated transdermal absorption of lidocaine hydrochloride
Source: PLoS One. 2026 Apr 3;21(4):e0344789. doi: 10.1371/journal.pone.0344789 (PMC13048389; doi:10.1371/journal.pone.0344789)
Supplement: Attachment — Submitted filename: Response_to_Reviewers_auresp_2.docx [file pone.0344789.s007.docx]

Response to Reviewers

Manuscript ID: PONE-D-25-63253

Title: Ionic-liquid-facilitated transdermal absorption of lidocaine hydrochloride

Dear Academic Editor and Reviewers,

Thank you for the opportunity to revise our manuscript and for the constructive comments provided by the reviewers. We have carefully addressed all points raised and have made substantial revisions to improve the manuscript. Our point-by-point responses are detailed below. All changes in the manuscript have been highlighted using the “Track Changes” function.

Response to Reviewer #1

We appreciate Reviewer #1’s positive evaluation of our revised manuscript. We are pleased that all previous comments have been adequately addressed and that the manuscript is now considered technically sound, statistically rigorous, and clearly presented. Thank you for your careful review and support.

Response to Reviewer #2

We sincerely thank the reviewer for the positive evaluation and constructive suggestions. We appreciate your recognition of our efforts in addressing the previous round of comments. Below we provide a detailed point-by-point response to the minor comments raised in this round of review.

Comment 1: You cannot claim that since the in vitro model is a mouse skin and not human model or in vivo study. Please rephrase this.

Response

We agree with the reviewer that conclusions regarding clinical waiting time should not be directly inferred from an ex vivo mouse-skin model. We have revised the corresponding statement in the Discussion to avoid clinical overinterpretation. The text now clarifies that the enhanced permeation observed within the early time window was obtained under experimental conditions using ex vivo mouse skin and does not directly predict clinical onset time. We have also added a statement emphasizing the need for validation in human skin models and in vivo studies.

The revised text reads as follows:

“Although rapid onset is desirable for topical anesthesia, our permeation data were obtained using an ex vivo mouse-skin model and therefore cannot be directly translated into clinical waiting time.”⸻

Comment 2: Statistics were only shown for the 5-hour time point. Please clarify whether statistical analyses were conducted at each time point.

Response

We thank the reviewer for this important comment. Statistical comparisons were performed at each individual time point using one-way ANOVA followed by Tukey’s multiple-comparisons test. The revised Figure 4 now reflects the statistical outcomes more comprehensively.

Specifically:

•	No significant difference was observed between the 50 mM and 80 mM groups up to 3 h (≤180 min), which is now explicitly indicated as “ns” in the figure.

•	Significant differences between the 50 mM and 80 mM groups emerged at 240 min (***p < 0.001) and 300 min (**p < 0.01).

•	At 300 min, both the 50 mM and 80 mM groups were significantly higher than the control group (***p < 0.001).

For clarity, only key time points are annotated in the figure, while statistical testing was conducted at all sampling times. The Results section (Section 3.4) and figure legend have been revised accordingly.

Comment 3: Improve clarity and organization of Introduction,Results and Discussion.

Response

We have revised Sections 1.(Introduction), 3.4 (Results) and 4 (Discussion) to improve logical flow and readability. In the Conclusions section, we reordered the presentation to follow the logical progression of cytotoxicity evaluation before permeation analysis, as suggested.

Comment 4: Minor spelling and typographical errors were noted.

Response

We thank the reviewer for carefully identifying minor spelling and typographical errors. All reported issues have been corrected in the revised manuscript. In addition, the entire manuscript has been thoroughly proofread to ensure consistency in terminology, grammar, and formatting.

We believe the revised manuscript has been significantly improved and now fully meets the publication standards of PLOS ONE. We are grateful for the insightful comments and hope you find our revisions satisfactory.

Sincerely,

Yangzhe Zhou, Yuqi Wen

On behalf of all co-authors
